# Software BreastAnalyser for the semi-automatic analysis of breast cancer immunohistochemical images

**DOI:** 10.1038/s41598-024-53002-6

**Published:** 2024-02-06

**Authors:** Marina Rodríguez-Candela Mateos, Maria Azmat, Paz Santiago-Freijanes, Eva María Galán-Moya, Manuel Fernández-Delgado, Rosa Barbella Aponte, Joaquín Mosquera, Benigno Acea, Eva Cernadas, María D. Mayán

**Affiliations:** 1 Institute of Biomedical Research of A Coruña (INIBIC), Complexo Hospitalario Universitario A Coruña (CHUAC), SERGAS, A Coruña, Spain; 2 CiTIUS - Centro Singular de Investigación en Tecnoloxías Intelixentes da USC, Universidade de Santiago de Compostela, Santiago de Compostela, Spain; 3 Department of Pathology, Complexo Hospitalario Universitario A Coruña (CHUAC), SERGAS, A Coruña, Spain; 4 Physiology and Cell Dynamics, Centro Regional de Investigaciones Biomédicas (CRIB) and Faculty of Nursing, Universidad de Castilla-La Mancha, Albacete, Spain; 5 Grupo Mixto de Oncología Traslacional UCLM-GAI Albacete, Universidad de Castilla-La Mancha, Servicio de Salud de Castilla-La Mancha, Ciudad Real, Spain; 6 Anatomic Pathology Unit, Hospital General Universitario de Albacete, Albacete, Spain; 7 Breast Unit, Complexo Hospitalario Universitario A Coruña (CHUAC), SERGAS, A Coruña, Spain; 8 CELLCOM Research Group. Biomedical Research Center (CINBIO) and Institute of Biomedical Research of Ourense-Pontevedra-Vigo (IBI), University of Vigo. Edificio Olimpia Valencia, Campus Universitario Lagoas Marcosende, 36310, Pontevedra, Spain

**Keywords:** Cancer, Cell biology, Biomarkers, Biomedical engineering, Cancer

## Abstract

Breast cancer is the most diagnosed cancer worldwide and represents the fifth cause of cancer mortality globally. It is a highly heterogeneous disease, that comprises various molecular subtypes, often diagnosed by immunohistochemistry. This technique is widely employed in basic, translational and pathological anatomy research, where it can support the oncological diagnosis, therapeutic decisions and biomarker discovery. Nevertheless, its evaluation is often qualitative, raising the need for accurate quantitation methodologies. We present the software BreastAnalyser, a valuable and reliable tool to automatically measure the area of 3,3’-diaminobenzidine tetrahydrocholoride (DAB)-brown-stained proteins detected by immunohistochemistry. BreastAnalyser also automatically counts cell nuclei and classifies them according to their DAB-brown-staining level. This is performed using sophisticated segmentation algorithms that consider intrinsic image variability and save image normalization time. BreastAnalyser has a clean, friendly and intuitive interface that allows to supervise the quantitations performed by the user, to annotate images and to unify the experts’ criteria. BreastAnalyser was validated in representative human breast cancer immunohistochemistry images detecting various antigens. According to the automatic processing, the DAB-brown area was almost perfectly recognized, being the average difference between true and computer DAB-brown percentage lower than 0.7 points for all sets. The detection of nuclei allowed proper cell density relativization of the brown signal for comparison purposes between the different patients. BreastAnalyser obtained a score of 85.5 using the system usability scale questionnaire, which means that the tool is perceived as excellent by the experts. In the biomedical context, the connexin43 (Cx43) protein was found to be significantly downregulated in human core needle invasive breast cancer samples when compared to normal breast, with a trend to decrease as the subtype malignancy increased. Higher Cx43 protein levels were significantly associated to lower cancer recurrence risk in Oncotype DX-tested luminal B HER2- breast cancer tissues. BreastAnalyser and the annotated images are publically available https://citius.usc.es/transferencia/software/breastanalyser for research purposes.

## Introduction

According to the latest GLOBOCAN estimates for 2020^1,2^, breast carcinoma is the most diagnosed cancer in both sexes worldwide (11.7%). It also accounts for the fifth cause of cancer mortality globally (6.9%) and the highest cancer-related mortality among women (15.5%). Breast cancer is a highly heterogeneous disease, comprising various profiles with different histopathological features detected by immunohistochemistry (IHC) analysis according to intrinsic molecular traits, clinical behaviours, and treatment responses. Traditional stratification follows the initial studies of Perou et al.^3^ and Sørlie et al.^4^, leading to the acknowledgment of five intrinsic molecular subtypes: luminal A, luminal B, HER2 overexpressing, basal and normal-like tumours. Luminal A breast tumours express estrogen receptor (ER) and/or progesterone receptor (PR), are negative for human epidermal growth factor receptor 2 (HER2), have low levels of protein Ki67 and are usually of grade 1 or 2. Their luminal B counterparts also express hormone receptor/s but some are positive for HER2; they are usually of higher grade (2–3) and express higher levels of Ki67 and other proliferation-related genes. Both luminal subtypes render the greatest outcome, although luminal A are linked to a significantly better prognosis. Both respond well to endocrine therapy and represent the most common breast cancer subtypes. HER2 enriched tumours are negative for hormone receptors and overexpress HER2 (*ERBB2*) and other genes in its amplicon such as *GRB7* and *PGAP3*. They are generally of high grade (2–3) and result in a poor outcome. They respond to available targeted agents, such as trastuzumab, an anti-HER2 monoclonal antibody. Basal-like/triple negative tumours show low expression or lack hormone receptors and HER2, and some patients/tumours express higher levels of proliferation-related genes and basal markers like keratins 5, 6, 14, 17 and epidermal growth factor receptor (EGFR). Usually of grade 3, they are defined by their aggressiveness, poorest prognosis, higher risk of relapse and metastasis, and lower disease-specific survival. This subtype has currently no targeted therapies available. Normal-like breast tumours share similar pathological markers as luminal A (ER+, PR+, HER2-, Ki67 low), show a normal breast tissue profiling, and result in an intermediate prognosis^3–5^. Regarding to this matter, it is important to draw attention to the grounbreaking multigene signature-based tests, such as Oncotype DX® (ODX; Exact Sciences, Madison, WI) that help guide clinical treatment decisions in ER+ HER2- lymph node negative early breast cancer^6^. A highly cost-effective test, it has been widely and clinically validated and it is the only gene-based assay certified to predict prognosis and benefit from chemotherapy treatment at the same time. Patients with scores between 0 and 25 present lower cancer recurrence risk if they undergo hormonal treatment, but are less likely to benefit from chemotherapy. Conversely, scores 26 and higher are indicative of high risk of cancer recurrence with hormonal therapy, whereas these patients can better benefit from adjuvant chemotherapy^7–9^.

In the context of breast anatomo-histopathology, 3,3’-diaminobenzidine tetrahydrochloride (DAB)-based IHC is a technique that stains specific protein antigens in brown, usually employing a cellular counterstain, such as hematoxylin, which renders the nuclei purplish blue. It has been regarded as a low cost yet highly complementary methodology, aiding in the diagnosis, subtyping and therapeutic indications of neoplasias. Another relevant application of IHC is related to the search of prognostic factors and biomarkers^10^. However, IHC assessment is often performed qualitatively and subjectively, such as presence/absence of target antigen, biasing its interpretation. Furthermore, apart from the degree of expression of a specific molecule, it is also crucial to discriminate its localization in various cellular compartments, such as the nuclei, which might be indicative of different biological roles^11^. Subsequently, there is a growing need for more accurate and reliable methods for IHC quantitation, with computer-based image analysis resulting in higher precision, solidity and quality in IHC quantification^12^.

Among the most popular tools in the biomedical field to analyse and quantify IHC images we find ImageJ^13^. It provides many common image processing algorithms and allows the definition of customized processing plugins. However, ImageJ does not allow object outlines to be corrected manually in a versatile and easy way before starting the image quantification. It is also worth mentioning Qupath^14^, an open source software for digital pathology image analysis. For DAB-IHC quantitation, Qupath offers a wide array of parameters that need to be fine-tuned in order to improve signal detection, resulting in a time-consuming process that requires certain expertise knowledge. Other recent approaches^15–21^ process automatically the IHC or histological images to detect and/or classify interest objects in the image, but they do not allow any expert supervision before the quantification, or require specific and expensive devices. We developed the CystAnalyser^22^ and STERapp^23^ software tools to quantitatively analyse histological images in medicine and biology, respectively. These tools overcome some limitations of ImageJ, QuPath and other approaches through a friendly graphical interface (GUI) easier to use for biomedical experts. Both softwares use image analysis and machine learning algorithms to automatically recognise and classify the objects of interest in the image, allowing the experts to review the recognition of objects using the GUI before measuring and counting them.

**Figure 1:**
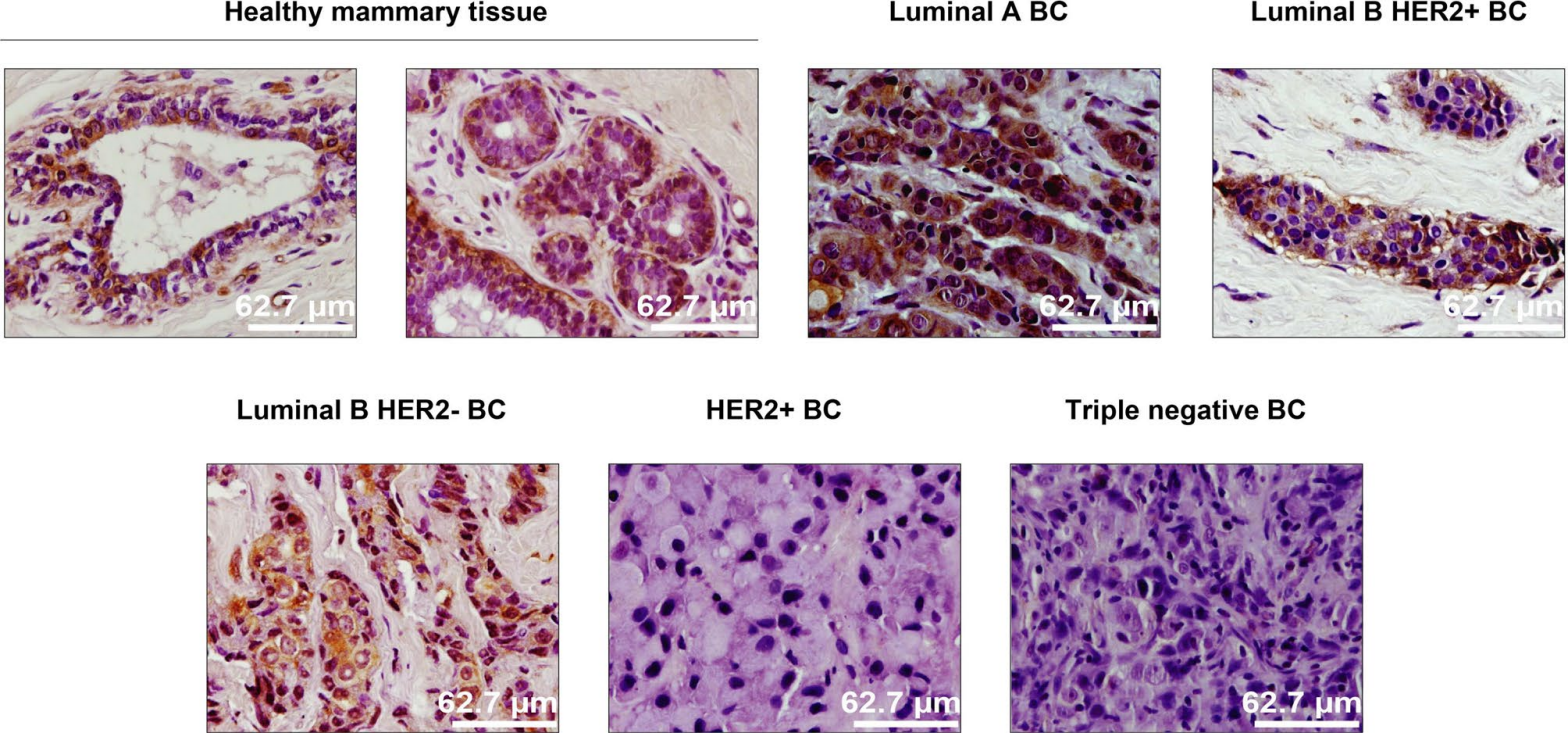
Representative IHC images against Cx43 (brown) and hematoxylin nuclear counter-stain (purple) of core needle biopsies of invasive breast cancer tumours not subjected to chemotherapy or radiotherapy, belonging to all 6 breast cancer subtypes: healthy mammary tissue, luminal A BC, luminal B HER2+ BC, luminal B HER2- BC, HER2+ BC and triple negative BC.

This paper proposes the software BreastAnalyser to quantify breast cancer immunohistochemical images of antigens relevant in breast anatomo-histopathology. In this work we focus, as a representative example, on the staining of the gap junction protein connexin43 (Cx43), in order to compare Cx43 levels among different subtypes and various degrees of breast cancer malignancy and risk of recurrence. Connexins (Cxs) are the basic protein components of the gap junction (GJ) channels and hemichannels, present in most cells and tissues. GJs allow direct communication between the cytoplasms of neighbouring cells, leading to the bidirectional passage of electrical signals and most small and soluble second messengers and molecules^24^. Connexins can also regulate several signalling pathways by their interaction with different protein partners^25^. Cx43 has been identified as a crucial component during mammary gland epithelial differentiation and development^26^. Loss of Cx43 and GJ functionality has been widely reported in breast tumour cell lines^27,28^ and primary tumours^27,29–31^. Nonetheless, the thorough involvement of Cx43 in the pathogenesis and development of breast cancer is far from being elucidated, hence more efforts are needed to comprehensively approach this topic.

BreastAnalyser is intended to fulfill the following requirements: (1) provide a friendly GUI to interactively work with the images; (2) use image analysis and machine learning algorithms to automatically recognise DAB-brown-stained proteins by immunohistochemistry, and detect and classify the nuclei in an image or ROIs; (3) automatically estimate various statistical measures and counts in the IHC images; (4) allow data sharing among researchers and review the results at any time; and (5) be fast enough to analyse images in real time. In relation to the previously mentioned softwares, BreastAnalyser represents a more straigthforward alternative, where specific post-processing concerns can be manually addressed straightway. BreastAnalyser, differently from QuPath, does not require modification of the annotations in order to correct mistakes regarding DAB-brown signal or nuclei detection/classification. Instead, BreastAnalyser allows an immediate adjustment of brown signal-pixels, using the add/delete/draw tools, and of located cell nuclei, by means of the add/delete/modify classification tools, with just a click of a button.

**Figure 2:**
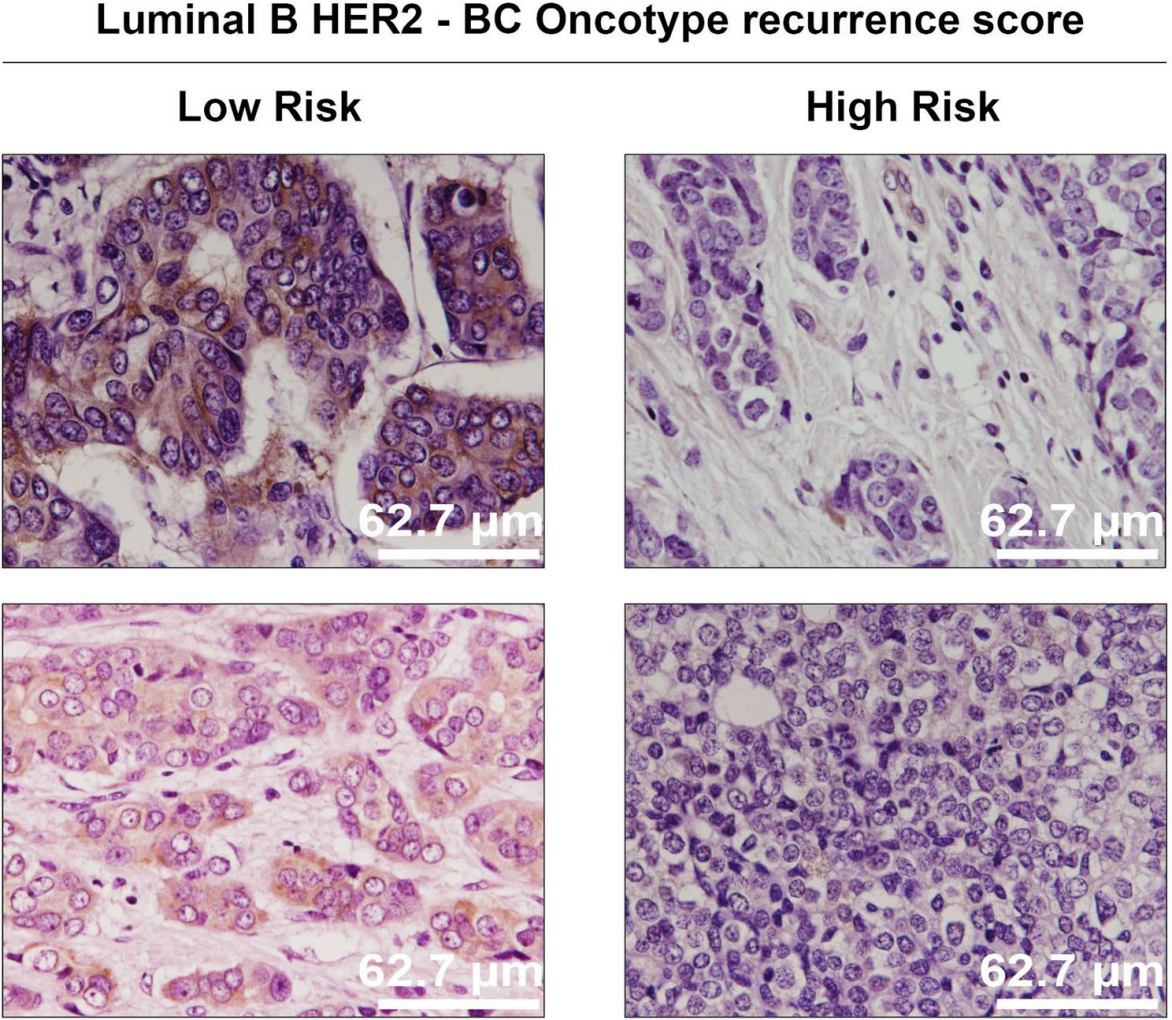
Representative Cx43-IHC images and hematoxylin nuclear counter-stain (purple) of invasive luminal B HER2- breast cancer tumours tested for Oncotype DX and categorized in low and high recurrence risk categories.

## Biological materials and experimental methodology

Biological samples used for Cx43 assessment comprise paraffin-embedded invasive breast tumours and normal mammary tissue controls, and are part of the A Coruña Biobank and our private collection of human biological samples. The samples used in our biomedical studies belong to white breast cancer female patients from the A Coruña sanitary area (NW Spain), with a median age of 52 years, being 48 % premenopausic and 52 % postmenopausic. Additionally, a variety of breast tumour immunohistochemistries were obtained from the collection of human biological samples of the Albacete General University Hospital in order to evaluate software inter-laboratory operation. Antigens detected are relevant in breast cancer anatomo-histopathological analysis and encompass RING1, RING4, CD99, CD31, CD177, EMA and cytokeratins 1 and 7. Immunohistochemistry was performed according to the standard protocols, described in detail in Section 1 of the supplementary material.

The IHC samples of INIBIC lab were photographed under an Olympus BX61 microscope coupled to an Olympus DP71 digital camera using a magnification of 40X, resulting in images of $2040\times 1436$ pixels. For the CRIB lab, images were acquired by a Nikon Eclipse 80i microscope coupled to a DXM1200C digital camera (Nikon), using magnification of 20*X* and 40*X*, resulting in images of $1372\times 1024$ pixels. One image was analysed from each sample-patient for the biomedical results discussion (“Biomedical results” section). The ROI were selected and manually drawn by the experts according to their anatomo-histopathological relevance (i.e.tumour cell nests or cords, healthy duct-lobular units and acini, etc).

**Figure 3:**
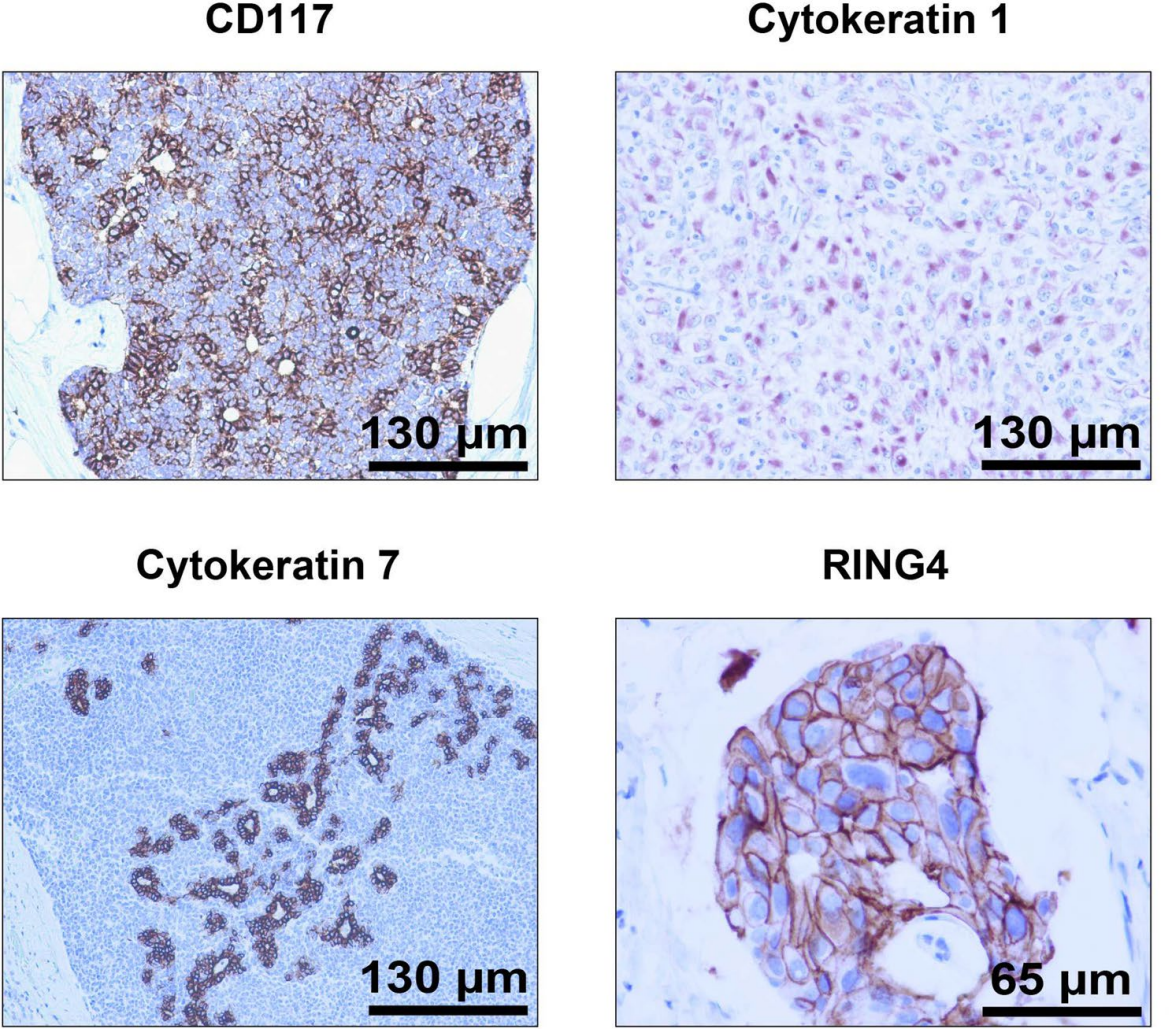
Representative IHC images from the CRIB lab.

We distributed the samples into three groups in order to perform different types of studies. The group **SET 1** is composed of 33 breast cancer patient samples derived from core needle biopsies of invasive tumours (grades 2–3) not subjected to chemotherapy or radiotherapy, in order to determine potential variations in Cx43 expression depending on the breast cancer subtype: luminal A (6 samples), luminal B HER2+ (5 samples), luminal B HER2- (8 samples), non-luminal HER2+ (7 samples) and triple negative (7 samples). This set also comprises 6 samples of normal healthy breast tissue for control. Specifically, **SET 1** includes 47 images extracted from the 39 samples, with Cx43 signal in brown, ranging from 0.06% to 38.3%. According to the expression and localization of Cx43 in human breast cancer (BC) tissues, it is progressively downregulated with increasing subtype malignancy. Figure 1 shows representative images of Cx43-IHC (brown) for each subtype, as well as for normal mammary tissue, where Cx43 immunostaining presents the highest expression levels, with membranous and cytoplasmic staining in both the myoepithelial and luminal layers of mammary ducts (1st panel) and acini (2nd panel). Luminal A breast cancer, the least aggressive subtype, is characterized by high Cx43 expression, mainly cytoplasmic (3rd panel), followed closely by luminal B HER2+ samples (4th panel). Luminal B HER2- tumours show lower Cx43 levels (cytoplasmic) (5th panel), whereas non luminal HER2+ (6th panel) and triple negative samples (last panel) score the lowest Cx43 levels, with almost no protein expression in the latter. **SET 2** comprises invasive breast cancer luminal B HER2- samples, grades 2–3, assessed for Oncotype DX Breast Recurrence Score Test: 6 scored high risk (scores 31–46) and 6 scored low risk (scores 9–20) for cancer recurrence. In this case, the purpose is to discern possible differences in Cx43 levels between high and low risk-scoring samples according to this test. Figure 2 shows representative Cx43-IHC images. It encompasses 32 images extracted from the 12 samples. **SET 3** contains 14 images extracted from 14 samples provided by the CRIB lab, which were purposely included for inter-laboratory software performance comparison. Figure 3 shows representative images of relevant antigens routinely assessed in breast cancer anatomopathological analysis.

## Computing methods

**Figure 4:**
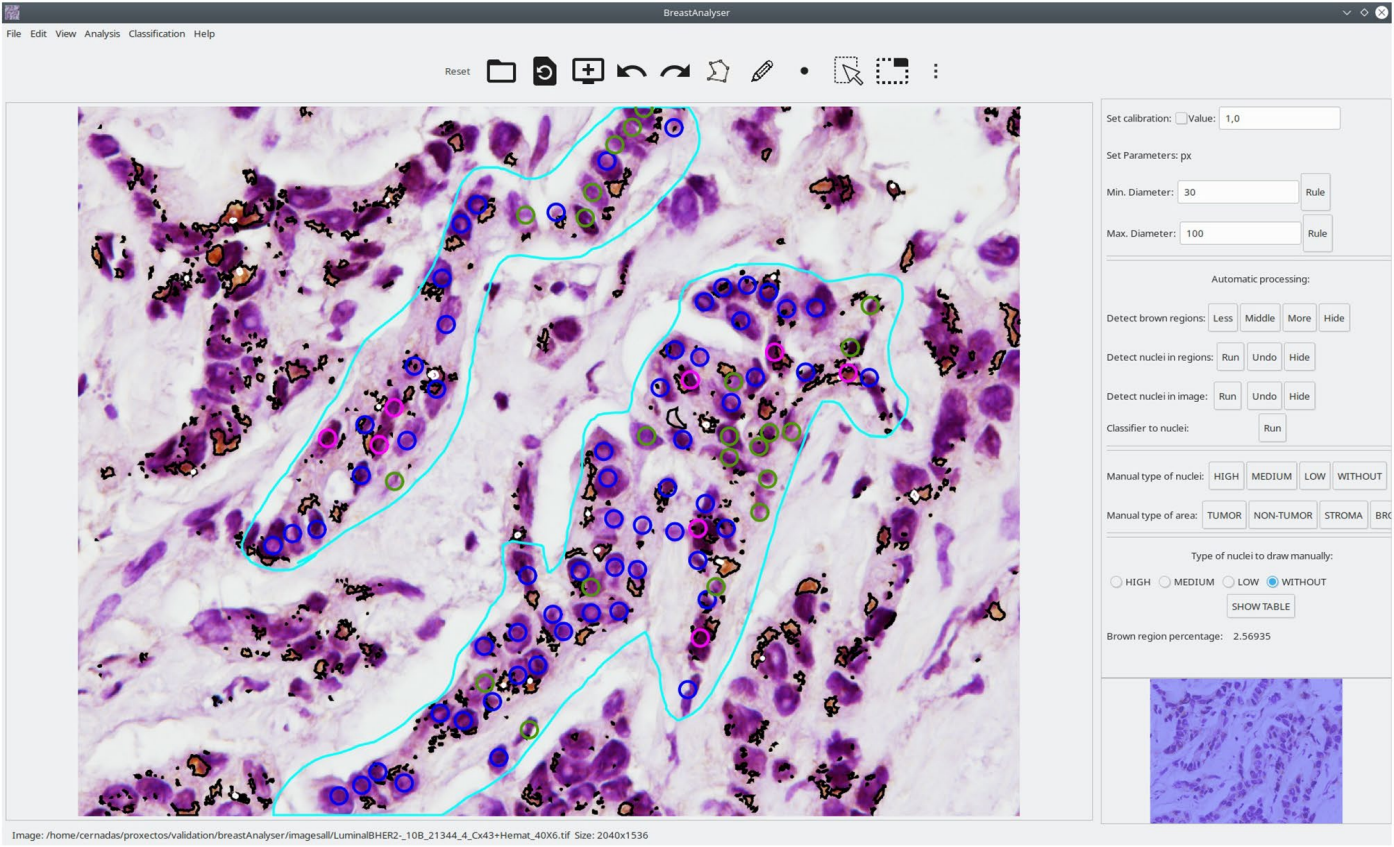
Screenshot of the BreastAnalyser GUI. In the region of analysis (defined by the cyan lines), the colour of the dots shows the category of the nuclei: yellow (highly stained), pink (moderately stained), blue (low stained) and green (not stained). The brown areas are surrounded by black lines, and the white regions are unstained zones inside the brown areas.

BreastAnalyser is a desktop application that runs on general purpose computers under Linux or Windows operating systems. It has been written in the C++ programming language using the GTK+ (GIMP Tool Kit) library (https://www.gtk.org/) to develop the GUI and the OpenCV library (https://opencv.org/) to generate the automatic image processing algorithms. Figure 4 shows its GUI with a typical IHC image loaded, processed and reviewed by the expert, and with the lateral panel displayed.

The “Functionality of BreastAnalyser” section describes the architecture and the main functionality provided by BreastAnalyser. In “Algorithms to recognise objects of interest” section details the image analysis algorithms to recognise the objects of interest (DAB-brown regions and nuclei) and “Machine learning methods” section describes the classification of the staining level of the nuclei.

**Figure 5:**
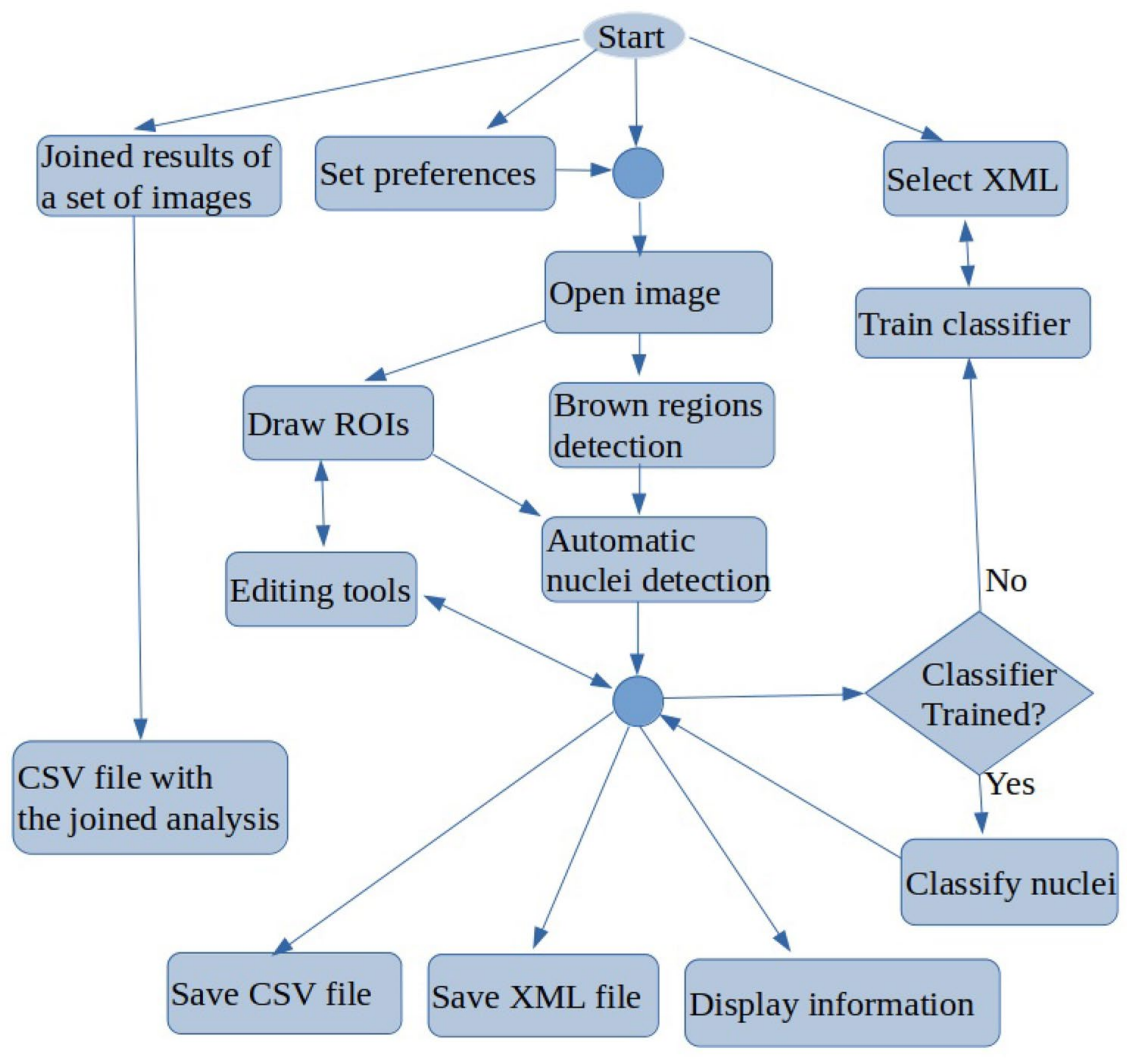
Flowchart containing the main tasks of BreastAnalyser.

### Functionality of BreastAnalyser

BreastAnalyser is a modular and extensible software composed by a GUI layer with editing tools to interact with the user; a logic layer that contains modules to automatically process the image and to calculate the statistical results; and a persistence layer to store all the data needed and calculated by the software. The software includes modules to: (1) store the image overlays, that contain the analysis supervised by the experts, in XML (Extensible Markup Language) files; and (2) save the statistical results, calculated from the overlays, in CSV (Comma-Separated Values) files.

Figure 5 shows a flowchart with the main functionality of BreastAnalyser. A typical working session for an user should have the following actions: (1) open an image; (2) automatically detect the DAB-brown regions; (3) automatically detect the nuclei in the image or into ROIs manually drawn and labeled by the expert as *TUMOR*, *NON-TUMOR* or *STROMA*; (4) automatically classify the detected or marked nuclei on the image; (5) go to expert’s supervision, as described below; (6) save the overlays drawn on the image into XML files; (7) export the statistical measures and counts to CSV files; and (8) at any time the user can set preferences, set calibration and diameters, save joined results of a set of images, or train the classifier.

Once the image is loaded, the buttons of the lateral panel can be used to automatically process the image following the instructions included in the user guide, provided as supplementary material. The detection and classification of the nuclei can be performed on the whole image or into ROIs drawn and labeled by the experts, depending on the objectives of the study. The classifier can be run in order to automatically provide a category for each detected nuclei. The available categories for the nuclei are “without staining” or “with low/medium/high staining”. Due to the inherent complexity of these images, the automatic processing may not be optimal for the expert, so BreastAnalyser provides an easy way to review the recognised objects in the images through the following editing tools (semi-automation): (1) delete a set of selected objects; (2) change the category of selected objects; and (3) add new nuclei specifying their category labels. The overlays set on the images contain their analysis information and they must be saved into the XML file in order to use other software functionalities, such as review the analysis, export joined results or train the classifier.

The working preferences of BreastAnalyser can be set going to the menu *File* $\rightarrow$ *Set preferences*, which allows to determine: (1) the working directories for images, overlays and results; (2) the width of points and lines; and (3) the colour of the overlays for each category. By default the measures on the images are provided in pixels. In order to obtain the results in real values, the user must set the spatial calibration, which is the relation between pixels in the image and real values (micrometers). The user must provide the minimum and maximum diameter of nuclei to be detected for an optimal operation of the automatic algorithms. These diameters can be set by writing in the *Preferences* dialog or graphically by drawing a straight line with the editing tools of the lateral panel. The preferences can be permanently stored for future working sessions. BreastAnalyser allows to export joined results of a set of images going to the menu *Analysis* $\rightarrow$ *XML Files*, which opens a dialog screen to select the XML files and the output CSV file. To do this task, the images have to be analysed and supervised by the expert, and finally the overlays must be saved in XML files, one per image. BreastAnalyser also allows to train the classifier going to the menu *Analysis* $\rightarrow$ *Train classifier*. More details of the BreastAnalyser use can be read in the user guide.

### Algorithms to recognise objects of interest

The recognition of DAB-brown or detection of nuclei on the image are segmentation problems where the DAB-brown regions or nuclei are the objects and the remaining area is the background. Image segmentation is an important topic in computer vision^32^, which is based on the analysis of the properties of homogeneity and discontinuity within the pixels of the image. Some image properties frequently used are colour, grey level or texture. The homogeneity paradigm develops the region detection algorithms, which try to keep the properties of the image constant within the regions. The discontinuity paradigm develops the edge-based algorithms, which attempt to find the position of the discontinuities in the image properties between the objects and the background. In this subsection, we describe the image segmentation algorithms included in BreastAnalyser. In “RBA to detect the nuclei” section describes the region-based BrownDetector algorithm to recognise the DAB-brown regions in the image. In BrownDetector algorithm to recognise DAB-brown signal” and “EBA algorithm to detect the nuclei” sections describe the region-based (RBA) algorithm and edge-based (EBA) algorithms used to detect nuclei. In the case of nuclei segmentation, experts only want to count the number of nuclei. So, the nuclei recognitions are transformed to points in BreastAnalyser. One of the main challenges to automatically process the pathology images is the colour variation among images due to differences in preparation and digitalization of samples, which can influence the performance of image analysis algorithms^33,34^. Normalization is often applied to original images in order to standardize them for processing^35^, but this pre-processing requires time and it might not be suitable for interactive applications. In this paper, we propose algorithms that are parametrized by the properties of each image. In this way the normalization is implicit in the algorithm itself, avoiding the normalization time.

**Figure 6:**
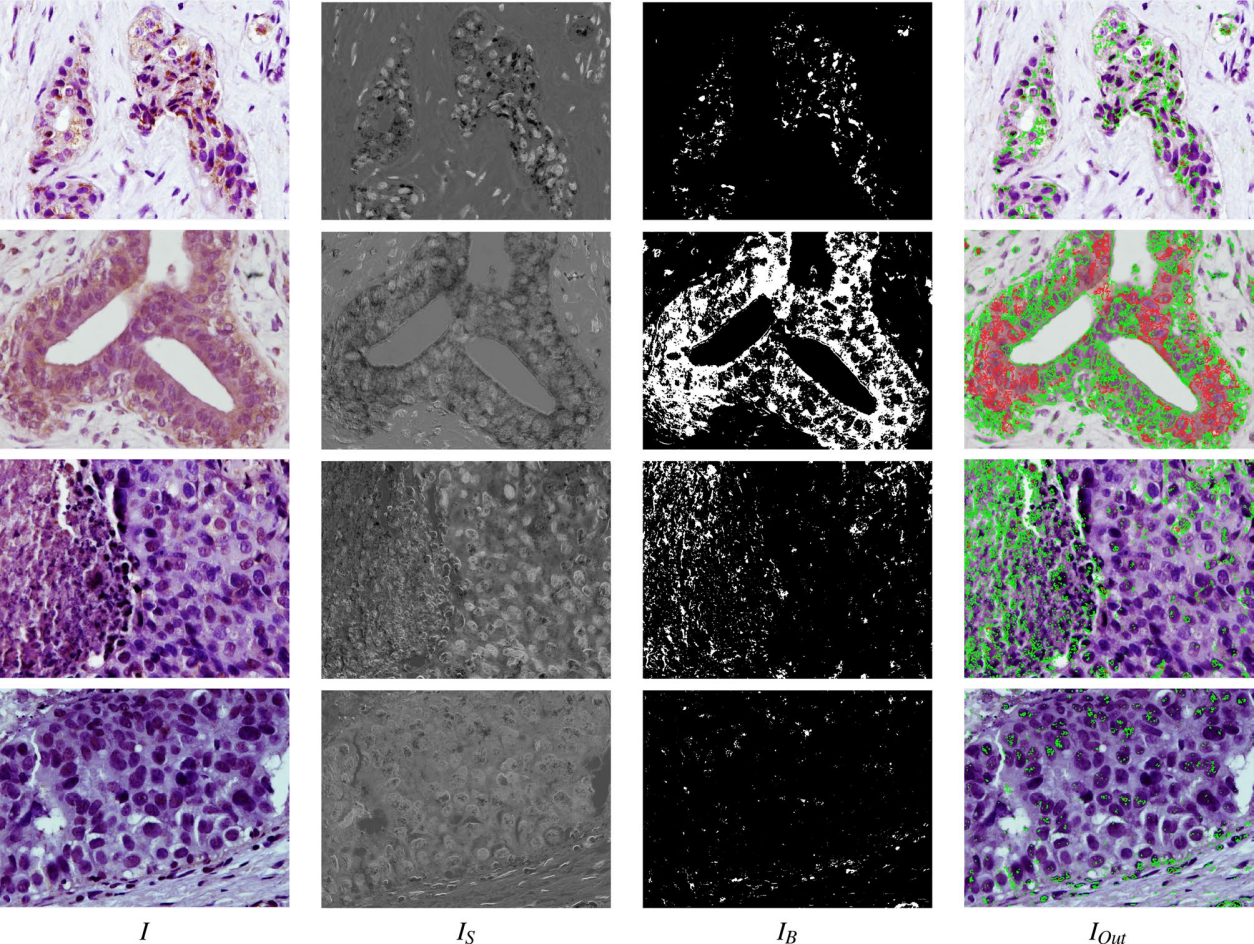
Examples of the automatic processing of IHC images using the BrownDetector algorithm for different types of processing: *I* is the original image, $I_S=I_aI_b$ being the $I_a$ and $I_b$ the *a* and *b* channel of *Lab* original image; and $I_B$ is the thresholded image (see the text for a detailed description). The $I_{Out}$ image shows the set of contours $\mathcal {R}$ (in green) and $\mathcal {H}$ (in red) recognised overlapped to the original image.

**Algorithm 1:**
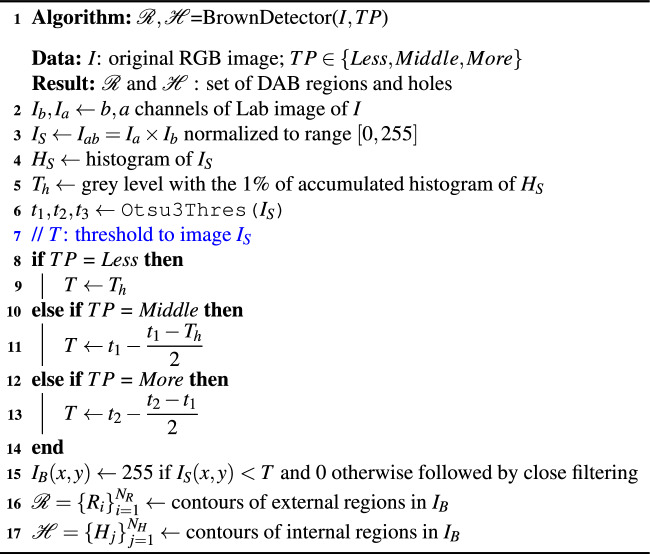
BrownDetector to recognise the DAB-brown regions.

#### BrownDetector algorithm to recognise DAB-brown signal

The proposed algorithm, called BrownDetector, to recognise the DAB-brown regions falls into the region based paradigm and it is a combination of computer vision techniques parameterized using the information of each image. The main steps of BrownDetector algorithm are summarized in the algorithm 1. Let *I*(*x*, *y*), with $x=1,\ldots ,N$, and $y=1,\ldots ,M$, be the original RGB image and let $TP=\{Less, Middle, More\}$ be the type of processing chosen by the expert, as it can be seen in the lateral panel of the GUI. Let $\mathcal {R}=\{ R_i \}_{i=1}^{N_R}$ be the set of $N_R$ DAB-brown regions automatically recognised, and $\mathcal {H}=\{ H_j \}_{j=1}^{N_H}$ be the set of $N_H$ regions, which represent holes inside the DAB regions. Firstly, the original RGB image *I* is transformed into the Lab colour space, that is more robust to illuminance variance^35^. Hence, the Lab colour space is intended to be perceptually uniform, i. e. changes in the numerical values are similar to the perceived change in colour. The *L* channel is associated to the lightness and the *a* and *b* channels are associated with the chrominance, specifically with the redness and yellowness respectively. In painting, the combination of red and yellow provides different shades of brown. So, the channels *a* and *b* are multiplied to build an image with different shades of brown developing the float image $I_{ab}$, which is transformed into the grey level image $I_S$ doing contrast stretching. In this image $I_S$, the lower values (darker pixels) correspond to the brownest pixels in the original image (see second column in Fig. 6 for visual examples). The $I_S$ image is thresholded in order to segment the foreground (DAB-brown regions) from the background. But, the selection of the optimal threshold value is a challenging task and many times it is determined by trial and error in the literature. In our approach, this threshold *T* is determined for each image from its statistical characteristics. Let $H_S$ be the histogram of image $I_S$, we define the threshold $T_h$ as the lowest grey level, starting from the 0, in the cumulative histogram in which the 1% of the total number of pixels in the image is achieved. Other thresholds are calculated using the multi-level method proposed by Otsu^36^, which selects a *T* that minimizes intra-class intensity variance maximizing inter-class variance. Specifically, let $t_1$, $t_2$ and $t_3$ be the thresholds calculated after applying the multi-level Otsu’s method with three thresholds to $I_S$ (function Otsu3Thres in algorithm 1). The optimal threshold *T* to binarize the image $I_S$ depends on the type of processing *TP* and it is calculated as $T=T_h$ when $TP= Less$; if $TP = Middle$, then $T = t_1 - (t_1 - T_h)/2$; and finally, if $TP = More$, then $T=t_2-(t_2-t_1)/2$. Normally, the best processing is achieved with the option $TP = Middle$, but the remaining options provide adequate responses to extreme cases, such as images with very low or great positivity. The image $I_S$ is transformed into a binary image using the threshold *T*, i.e. if $I_S(x,y) < T$ the output is 255 and 0 otherwise. This process is called inverse thresholding. Mathematical morphology is commonly used for morphological processing of images, which is composed by the basic filters: dilation, erosion, open and close. Initially, it was defined to binary images in order to fill/remove objects smaller than the size of structural element^32^. So, we use a close filter with masksize 5 to fill small holes in the thresholded images developing the image $I_B$. The contours of the DAB-brown regions (set $\{ R_i \}_{i=1}^{N_R}$) and holes into DAB-brown regions (set $\{ H_j \}_{j=1}^{N_H}$) are extracted from $I_B$ using the algorithm proposed by Suzuki and Be^37^. The number of DAB regions is the dimension of the set $\mathcal {R}$ and the DAB-brown area percentage is calculated adding up the area of regions in set $\mathcal {R}$ and subtracting the area of regions in $\mathcal {H}$. Figure 6 shows visual examples of the performance of BrownDetector algorithm in different breast cancer samples using green colour for DAB regions and red for holes inside DAB regions: (1) Luminal B HER2+ (top row): using the option *Middle*, the threshold *T* calculated is 54 and the percentage of DAB area is 2.19%; (2) healthy breast (second row) using option *More*, $T=93$ and the 27.4% area occupied by DAB regions; (3) triple negative (third row) using the option *Middle*, $T=62$ and 5.59% of DAB area; and (4) HER2+ (bottom row), using option *Less*, $T=69$ and 0.93% of DAB area.

#### RBA to detect the nuclei

**Algorithm 2:**
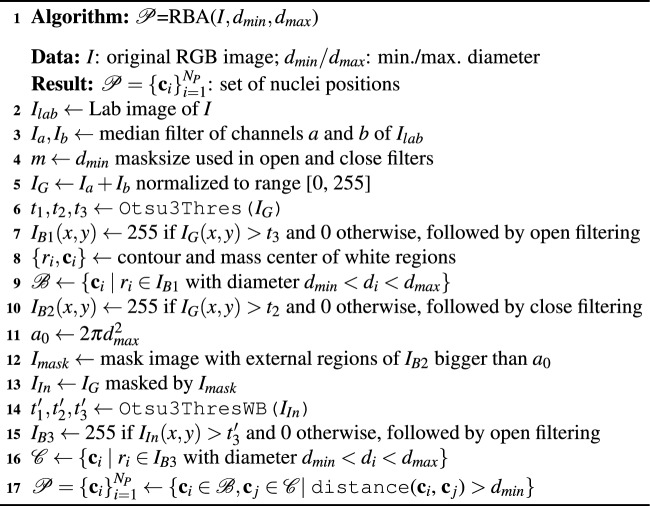
RBA algorithm to detect nuclei.

**Figure 7:**
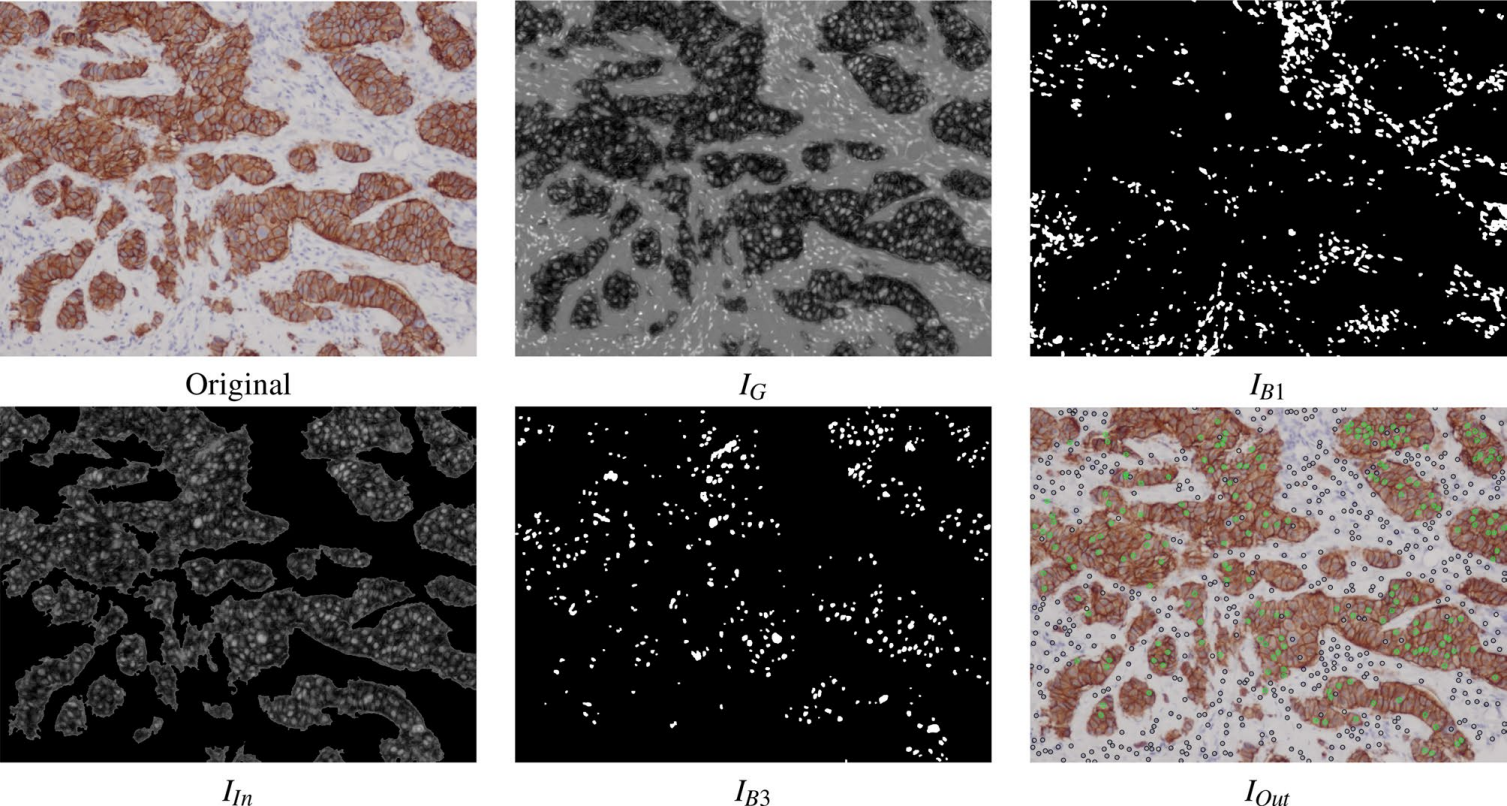
Examples of the automatic processing of immunohistochemical images using RBA to detect the nuclei (see algorithm 2 for the meaning of $I_G$, $I_{B1}$, $I_{in}$ and $I_{B3}$). The image $I_{Out}$ shows the set of nuclei positions $\mathcal {P}$ overlapped to the original image (in black and green the nuclei without staining and low staining, respectively, using the classifier).

The RBA algorithm attempts to segment the nuclei using the region-based segmentation paradigm. Afterwards, the mass center of each segmented region is considered as the detection of each nucleus. The main steps of RBA are summarised in algorithm 2. Let *I* be the original RGB immunohistochemical images and $d_{min}$ and $d_{max}$ the minimum and maximum diameter of the nuclei to be detected. Firstly, the *I* image is transformed to the Lab colour space and the colour *a* and *b* channels, $I_a$ and $I_b$ respectively, are considered, which are processed by a median filter with masksize 3 in order to attenuate random noise. Secondly, $I_G$ is built by adding the $I_a$ and $I_b$ images and normalizing the result to the range [0,255] (see a visual example of the $I_G$ image in Fig. 7). The nuclei appear as bright spots inside the background, but the brightness of the nuclei outside the DAB-brown regions is higher than that of the inner ones. So, we detect the nuclei using two stages: (1) threshold $I_G$ image using $t_3$ to detect the nuclei outside of the DAB-brown regions; and (2) create a mask image, $I_{mask}$, containing only the DAB regions and apply thresholding to detect the nuclei inside the DAB regions. In all cases, the threshold values are determined automatically using the multi-level method of Otsu^36^, function Otsu3Thres and Otsu3ThresWB in algorithm 2. In the first step, the optimal threshold $t_G$ to segment the $I_G$ images is the third threshold ($t_3$ in algorithm 2, $t_3=132$ in Fig. 7). The resulting image after thresholding is processed by a morphological open filter using a masksize $m=d_{min}$ in order to remove objects smaller than the structural element and split touching nuclei, developing the $I_{B1}$ image (see Fig. 7). The set of nuclei positions, $\mathcal {B}$, includes the mass centers of the white regions in $I_{B1}$ with diameter *d* such that $d_{min}<d<d_{max}$.

In the second step, the value $t_2$ (line 6 of algorithm 2) is used to inverse threshold $I_G$ developing the image $I_{bin}$ (for example in Fig. 7 we use $t_2=88$). The $I_{bin}$ image is post-processed by a close filter of size *m* in order to fill holes smaller than the nuclei size. We only keep the external regions larger than an area $a=2\pi d_{max}^2$ to create a mask image, $I_{mask}$, which multiplied by the $I_G$ image develops the $I_{In}$ image in Fig. 7. In these images the DAB-brown regions are seen, while the remaining dark areas in image are associated to background. We apply again the multi-level Otsu method to the $I_{In}$ image without considering the black pixels (function Otsu3ThresWB in line 14 of algorithm 2) and use the third value $t'_3$ to threshold the $I_{In}$ image ($t'_3=89$ for example in Fig. 7). The $I_{B3}$ image in Fig. 7 shows the resulting image after applying an open filter with masksize *m* to remove noisy regions. The set $\mathcal {C}$ of nuclei positions is obtained by applying the size filter to the white regions in image $I_{B3}$ (i.e. regions whose diameter *d* satisfies $d_{min}<d<d_{max}$, line 16 of algorithm 2). Finally, we apply an overlapping test to remove the nuclei which have been detected by the two stages. So, $\mathcal {P}=\{ \textbf{c}_i \}_{i=1}^{N_P}$ will be the nuclei that are farther from each other than $d_{min}$, i.e. $\{ \textbf{c}_i \in \mathcal {B}, \textbf{c}_j\in \mathcal {C} |$ distance($\textbf{c}_i$, $\textbf{c}_j$) $> d_{min} \}$. The $I_{out}$ image in Fig. 7 shows the original image with the nuclei positions, set $\mathcal {P}=\{ \textbf{c}_i \}_{i=1}^{N_P}$, overlapped as circles for visualization purpose. The colour means the staining level of the nuclei (black and green for non-stained and low stained nuclei, respectively).

**Algorithm 3:**
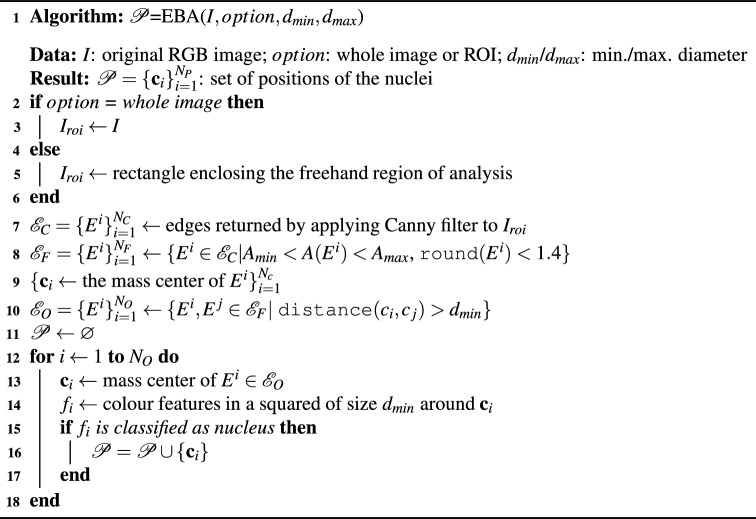
EBA algorithm to detect nuclei.

#### EBA algorithm to detect the nuclei

The EBA algorithm to detect nuclei is based on the edge-based paradigm. Specifically, EBA is a modified version of the Canny filter, proposed by Canny^38^, already used in our software Govocitos^39^. Algorithm 3 summarizes the EBA algorithm, that only uses one Canny filter tuned with a Gaussian smoothing width $\sigma =4$ in order to remove image noise. The thresholds of the hysteresis process in the Canny filter are automatically calculated from the image characteristics using rates of 0.4 and 0.6 for the lower and higher thresholds, respectively. The output of the Canny filter is a set of edges $\mathcal {E}_C=\{ E^i \}_{i=1}^{N_C}$, that is post-processed as follows to calculate the positions of nuclei. The set $\mathcal {E}_C$ is filtered using the minimum ($d_{min}$) and maximum ($d_{max}$) diameters for the nucleus provided by the expert. If we considered that the nucleus is rounded, its area can be approximated by a circle with area $A=\pi d^2/4$, being *d* the diameter of the nucleus edge $E^i$. So, we use the following criteria: (1) the area $A(E^i)$ enclosed by $E^i \in \mathcal {E}_C$ satisfies that $A_{min}< A(E^i) < A_{max}$, being $A_{min}$ and $A_{max}$ the areas calculated using $d_{min}$ and $d_{max}$, respectively; and (2) the roundness of edge $E^i$, (round function in algorithm 3) is lower than 1.4, since the circle roundness is 1. This post-processing filtering creates a new set $\mathcal {E}_F= \{ E^i \}_{i=1}^{N_F}$ with $N_F \le N_C$. To avoid two detections of some nuclei, an overlapping test is applied: an edge $E^i \in \mathcal {E}_F$ is removed if there is another edge $E^i$ with $\texttt {distance}(\textbf{c}_i, \textbf{c}_j) \le d_{min}$. After this overlapping test, a new set $\mathcal {E}_O=\{ E^i \}_{i=1}^{N_O}$ with $N_O \le N_F$ is created. Finally, we use a pre-trained classifier to predict if an edge $E^i \in \mathcal {E}_O$ is a true nucleus or a false positive (see the “Machine learning methods” section for details). The input of the classifier is a numerical vector including the mean values of the Lab colour space channel in a square of size $d_{min}$ centered in the centroid $\textbf{c}_i$ of edge $E^i \in \mathcal {E}_O$. If the classification prediction is a nucleus, the centroid is added to the set of nuclei $\mathcal {P}=\{ \textbf{c}_i \}_{i=1}^{N_P}$. In order to process an user-defined ROI, the EBA algorithm is applied only on a rectangle enclosing the ROI. Examples of the visual performance of the EBA algorithm can be seen in the user guide.

### Machine learning methods

A classifier is a machine learning method for the automatic prediction of discrete values (output categories) based on data examples. The classifier learns to predict the outcome category as a function of the input data in a process called “training”, that uses a collection of examples, each composed by the input data and the outcome value. During training, the model changes the values of its parameters in order to predict an outcome near to the true value for the training data, i.e., to give a reliable prediction for these data. The trained model is expected to generalise its predictions with reliability to new input data not used during training.

BreastAnalyser includes two classifiers: ClassifierFP, used to discriminate between valid (true-positive) and non-valid (false positive) nuclei; and ClassifierNucleus, used to discriminate among different staining levels in the nuclei: with high, medium or low staining and not stained. Both classifiers use the support vector machine (SVM) with radial basis function (RBF) kernel, selected because it is one of the best-performing machine learning models for classification^40^. Specifically, BreastAnalyser uses the LibSVM implementation^41^, accessed through its C++ binding. The SVM used by ClassifierNucleus for the staining level of the nuclei can be re-trained from the GUI at any time through the submenu *Classification* $\rightarrow$ *Train classifier*. In this case, the SVM is trained using a collection of nuclei randomly selected from the XML files provided by the user. In order to avoid an excessively slow training, a maximum number of 1,000 nuclei are selected. Whenever posible, the nuclei are selected in similar numbers, requiring a minimum number of 10 nuclei, for each staining category. BreastAnalyser performs the tuning of the two hyper-parameters of the SVM (regularization $\lambda$ and RBF kernel spread $\sigma$) using the grid-search method. The performance is evaluated by the Cohen kappa statistic^42^, which measures the coincidence between the true and predicted category excluding the agreement by chance. Kappa (in %) is defined as:

1 $$\begin{aligned} kappa=100\frac{p_a-p_e}{s-p_e}, \qquad p_a=\displaystyle \sum _{i=1}^C N_{ii}, \qquad p_e=\displaystyle \frac{1}{N^2}\sum _{i=1}^C \left( \sum _{j=1}^C N_{ij} \right) \left( \sum _{j=1}^C N_{ji} \right) , \quad s=\displaystyle \sum _{i=1}^C \sum _{j=1}^C N_{ij} \end{aligned}$$

where $N_{ij}$ is the number of nuclei of category *i* that are assigned by the SVM to category *j*, while $C=4$ is the number of categories and *N* is the number of nuclei. The values of $\lambda$ and $\sigma$ used for hyper-parameter tuning are: $\lambda =\{ 2^{2i-7} \}_{i=1}^{10}$ and $\sigma =\{ 2^{-(i+1)/2} \}_{i=-15}^0$. For each combination of hyper-parameter values, the SVM is trained using the *K*-fold cross-validation methodology with $K=4$, so that $K-1=3$ folds are used to train the SVM, and the remaining fold is used to calculate the kappa of the trained SVM. The training and prediction are performed *K* times, rotating the folds each time (i.e., in the first trial folds 1-3 are used for training and fold 4 for test; the second trial uses folds 2-4 to train and fold 1 for test; and so on) and averaging kappa over the *K* test folds. The process is repeated for all the combinations of hyper-parameter values, and the one that achieves the highest average kappa is selected. Finally, the SVM is trained over the whole collection of nuclei, using the selected combination of hyper-parameter values, and then it is ready to predict the category for new nuclei.

The default ClassifierNucleus included in BreastAnalyser was trained with a selected set of samples provided by INIBIC lab composed by 26 IHC images (from patients not included in **SET 1** nor **SET 2**) containing 1,359 nuclei with different staining levels: 160 high, 195 medium, 328 low and 676 nuclei without staining, selected attempting to represent all the posible variability in the staining level of the nuclei. The training followed the methodology described in the previous paragraph.

The feature vector used by both SVM classifiers contains the mean value of each channel in the Lab image over a neighborhood of the nucleus. This neighborhood is centered on the centroid of the nucleus and its size is equal to the minimum diameter of nuclei, fixed by the expert using the BreastAnalyser GUI.

## Results

BreastAnalyser has been used since 2021 in the daily research work of experts at INIBIC lab in order to evaluate the software in a real environment. Since 2022, this software is also being used in CRIB lab in order to assess inter-laboratory operation. The interaction of clinical staff with BreastAnalyser was logged into the XML files to perform a statistical evaluation of the automatic algorithms of image processing and machine learning included in the software. The main goal of this research is the evaluation of the robustness and versatility of BreastAnalyser software when exposed to highly variable IHC images, representative of the complexity and inter-patient variability of a real-world clinical scenario. Even though the number of samples analysed is limited (57, 32 and 14 images for data set 1, 2, and 3 respectively), due to availability constraints, they can faithfully recapitulate the multifactoriality of the disease. In the following, we describe the statistical measures used to evaluate the automatic processing algorithms (Sectïon "Statistical measures"), show the DAB-brown signal quantification results (Section "Automatic recognition of DAB-brown signal"), present the results of detection (Section "Detection of nuclei") and classification (Section "Classification of nuclei") of nuclei and discuss the software experts’ perception and performance (Section "Elapsed time and system usability"). Finally, we address the relevance of the obtained Cx43 measurements in the context of breast cancer biomedical research (Sectïon "Biomedical results").

### Statistical measures

The statistical evaluation of the algorithms to automatically recognise the objects of interest (DAB-brown regions or nuclei) can be measured counting the number of hits and mistakes in the detection. We consider that a detection is a true positive (TP) hit when the user did not modify the automatic recognition provided by the algorithm; a false positive (FP) when the user manually deleted the objects provided by the computer; and a false negative (FN) when the user manually added a DAB region/nucleus.The number of DAB regions/nuclei automatically recognised is defined by the sensitivity (Se) or recall (R), positive predictivity value (PPV) or precision (P), average precision and $F_1$-score, all in %:

2 $$\begin{aligned} Se =R= \displaystyle 100 \frac{TP}{FN + TP}, \quad PPV =P= \displaystyle 100 \frac{TP}{FP + TP}, \quad AP= \displaystyle 100 \frac{TP}{TP + FP + FN}, \quad F_1=\displaystyle 100\frac{2PR}{P+R} \end{aligned}$$

In the case of positivity estimation, the BrownDetector algorithm can be evaluated measuring the area percentage occupied by DAB-brown regions reported by the computer (APC) and after the expert’s supervision (APES). The difference $|APC-APES|$ provides the computer error for estimating the image positivity.

The performance of the SVM model in the prediction of the nuclei category $\mathcal {C} \in${highly stained, medium stained, low stained, no stain} is evaluated using the Cohen kappa, defined in Eq. 1 above, and the accuracy (in %), whose value is 100 multiplied by the number of nuclei correctly classified by the classifier ($p_a$ in the Eq. 1) and divided by the total number of nuclei. The $Se_i$ and $PPV_i$ of each category $i\in \mathcal {C}$ are also calculated considering that: (1) the $TP_i$ are the number of nuclei of category $C_i$ correctly classified by the SVM into the category $\mathcal {C}_i$; (2) the $FP_i$ are the number of nuclei classified into category $\mathcal {C}_i$, but whose true category label is other; (3) the $FN_i$ are the number of nuclei of true category $\mathcal {C}_i$ that the classifier assigned to other category; and (4)$TN_i$ (True Negative) is the number of nuclei of true category $\mathcal {C}_j$, $j\ne i$, classified by SVM as any category $\mathcal {C}_j$, $j\ne i$. The specificity ($Sp_i$) is defined as:

3 $$\begin{aligned} Sp_i=\displaystyle 100\frac{TN_i}{TN_i+FP_i} \end{aligned}$$

### Automatic recognition of DAB-brown signal

Table 1 shows the results of the BrownDetector algorithm (BrownDetector algorithm to recognise DAB-brown signal section) to automatically recognise the DAB-brown regions in the IHC images, while APC and APES are the area percentages automatically detected by the computer and after the expert’s supervision, respectively. In all data sets, the sensitivity for recognizing DAB-brown areas is higher than 99% and the positive predictivity value is higher than 95%. Hence, the $F_1$-score achieves values higher than 96% for all data sets. The difference between the area percentage recognised automatically by the computer and after expert’s supervision is only 0.07 and 0.05 points for SET 1 and SET 2 respectively. Hence, the experts only supervise the 10% and 28% of images for SET 1 and SET 2 respectively. But, it is important to emphasise that the maximum difference of area percentage was only of 1.5 and 1.67 points for SET 1 and 2 respectively, i.e. the area of the DAB-brown regions corrected by experts was very low and affects to very small regions. For the SET 3, the software operates perfectly (precision of 100%). This good performance of the automatic BrownDetector algorithm leads us to conclude that the system could be used in routinary tasks of biomedical labs.

**Table 1:** Sensitivity (Se), positive predictivity value (PPV), average precision (AP) and $F_1$-score, in %, of BreastAnalyser working in the lab to recognise the DAB-brown regions in IHC images. The APC and APES are the area percentages detected automatically by the computer and after the expert’s supervision, respectively.

Data set	#images	Se	PPV	AP	$F_1$	APC	APES	|APC-APES|
**SET 1**	47	99.2	95.5	95.3	96.1	9.13	9.06	0.07
**SET 2**	32	99.8	95.4	95.1	97.2	2.68	2.66	0.05
**SET 3**	14	100.0	100.0	100.0	100	22.92	22.92	0.00

### Detection of nuclei

**Figure 8:**
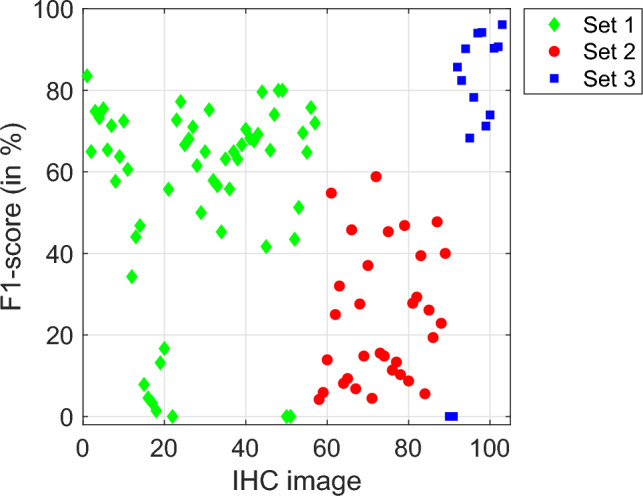
$F_1$-score (in %) for each image in SET 1 (green diamonds), SET 2 (red circles) and SET 3 (blue squares).

Table 2 shows the statistical results provided by RBA and EBA (see “RBA to detect the nuclei” and “EBA algorithm to detect the nuclei” sections, respectively) to automatically detect the nuclei in IHC images. The sensitivity is quite different among sets (70.4% and 48.2% for SET 3 and 1 respectively and only 16.6% for SET 2), which means that the algorithms detect much more true nuclei in the SET 3. Nevertheless, the positive predictivity value is quite similar and high for all sets (about 70%), denoting that the algorithms do no spot many false positive nuclei. The average precision includes both types of errors, misdetections and false detections, but it is dominated by the sensitivity behaviour (63.8% for SET 3, 41.4% for SET 1 and 14.7% for SET 2). This behaviour is also observed in $F_1$-score, which ranged between 24.1% for SET 2 and 96.1% for SET 3. Figure 8 shows the $F_1$-score for nuclei detection for all images. Although the nuclei detection in SET 3 is much higher than in the remaining data sets, a high variability among images can be observed in all data sets, ranging from 0% (i.e. the algorithm does not detect nuclei or detects many false nuclei, so a better option is to manually detect the nuclei using a mouse click) up to $F_1$=96%. The nuclei detection algorithms work rather poorly when the nuclei are partially masked by the DAB-brown signal.

**Table 2:** Sensitivity (Se), positive predictivity value (PPV), average precision (AP) and $F_1$-score, in %, of BreastAnalyser for the nuclei detection on each set. The value N reports the average number of nuclei per ROI or image.

Data set	#images	#ROI	N	Se	PPV	AP	$F_1$
**SET 1**	47	58	55	48.2	71.8	41.4	55.2
**SET 2**	32	32	50	16.4	66.2	14.7	24.1
**SET 3**	14	13	45	70.4	76.5	63.8	96.1

### Classification of nuclei

In order to evaluate the classification of the nuclei according to their level of staining (**high**, **medium**, **low** and **no** staining), an expert assigned a category to all the nuclei in the ROIs studied and, subsequently, the expert’s labels were compared with the labels provided by the classifier. Then, the default pre-trained classifier included in the BreastAnalyser software was used to predict the category label. The classification accuracy and kappa evaluate the agreement between both category labelings. In **SET 1**, accuracy and kappa are 48.9% and 18.2%, respectively. The confusion matrix is reported by Table 3. The value in row *i* and column *j* is $100N_{ij}/N$, where $N_{ij}$ and *N* are the same as in Eq. 1 above. The diagonal numbers (in bold) give the percentage of nuclei correctly classified for each category, and the sum of the diagonal gives the classification accuracy. In general, the percentages in the main diagonal are higher than the percentages outside it, meaning that the classifier can discriminate the categories. Nevertheless, for some categories (e.g. low and no stained) the percentages are quite similar, which explains the relatively low values of accuracy, sensitivity and predictive positive value. The specificity is quite high for high and medium stained (above 92%) and moderated for low and no stain (about 60%). The staining level of the nuclei is a continuous property, so it is possible that the subjectivity of the experts or the non-linear responses of the human visual system label the nuclei depending on their surroundings. But, it is important to note that the classification is almost always confused with the neighbouring category (i.e., low and no staining). Hence, if we consider as success when the classifier predicts the true or the contiguous category, the classification accuracy is increased up to 95.59%. In line with this, if our main objective in the biomedical part of this work is to discriminate between weak and strong staining, as is customary in the literature, and if we consider only two classes (weak=no+low, strong=medium+high), then the accuracy is 89.32%.

**Table 3:** Confusion matrix (in %) for nuclei staining classification (high, medium, low and without staining) for **SET 1**. The columns Se, PPV and Sp report the sensitivity, predictive positive value and specificity for each class, respectively.

			Predicted category						
		#Nucleus	High	Medium	Low	No	Se	PPV	Sp
	High	51	**0.94**	0.54	0.07	0.17	54.90	34.15	98.2
True	Medium	327	1.27	**5.53**	1.81	2.35	50.46	46.87	92.9
Category	Low	1638	0.50	4.33	**23.47**	26.63	42.73	62.72	69.0
	No	967	0.03	1.41	12.07	**18.88**	58.28	39.31	57.0

In the **SETS 2** and **3** the classification was evaluated within the routinary use of BreastAnalyser. After drawing the ROIs, the automatic detection of nuclei is executed and classification is performed on the nuclei detected automatically. Afterwards, the expert supervised jointly the detection and classification results. In these experiments, the accuracy was 100% for the SET 2 and 93% for SET 3. This accurate classification of the staining level can be explained by two reasons: (1) the expert did not change the nuclei labels; and (2) the correctly detected nuclei are not partially masked by other types of tissues, and then, in these cases, the classifier predicts correctly the true category.

### Elapsed time and system usability

The elapsed time for the analysis of an IHC image depends mainly on the time required to review the automatic recognition of DAB-brown areas and the nuclei detection. This values were estimated by the INIBIC lab experts using a stopwatch to measure the time needed to load, process, review and save the results for each image on a standard personal computer with 4 cores AMD Ryzen 5 3500U at 2.10 GHz and 8 GB of RAM memory under Windows operative system. The average elapsed time estimated was 3.75 minutes. Obviously, the analysis time is dominated by the review time. So, in order to estimate the time spent by the automatic processing, additional experiments were done on a computer with 8 Intel® Core^TM^ i7-9700K CPUs at 3.60 GHz and 64 GB of RAM memory under Linux Kubuntu 20.04. The average time spent to process an image of $2040\times 1436$ pixels was 0.43 miliseconds to BrownDetector algorithm, 0.49 miliseconds to EBA and 0.16 miliseconds to RBA.

The subjective expert perception and system usability was evaluated using the System Usability Scale (SUS) questionnaire^43^, which measures the learning abitity and perceived usability of the software. The SUS has 10 items with a five-point scale. If the score is below 25 it is the worst imaginable system; between 25 and 39 is “from worst imaginable to poor”; between 40 and 52 is “from poor to OK”; from 52 to 73 is “OK to good”; from 73 to 85 is “good to excellent” and from 85 to 100 is “excellent to best imaginable”^44^. Ten experts from the participating biomedical laboratories belonging to clinical pathology and biomedical research areas filled out the questionnaire and gave an average score of 85.5 points, ranging from 67 to 97.5 points. This result means that BreastAnalyser is perceived as excellent.

### Biomedical results

BreastAnalyser was used to quantitate Cx43 immunostaining in various invasive breast cancer tissue samples, representative of the complexity and inter-patient variability of clinical specimens. This study was performed for the first two data sets: (1) **SET 1** to analyse potential variations in Cx43 expression depending on the breast cancer subtype; and (2) **SET 2** to discern possible differences in Cx43 levels in invasive breast cancer luminal B HER2- tissues according to their Oncotype DX Breast Recurrence Score Test results (Figs. 1 and 2 show representative IHC images). All images of **SET 1** and **SET 2** were automatically analysed and supervised, semi-automatically, by an histopathological expert using BreastAnalyser.

**Figure 9:**
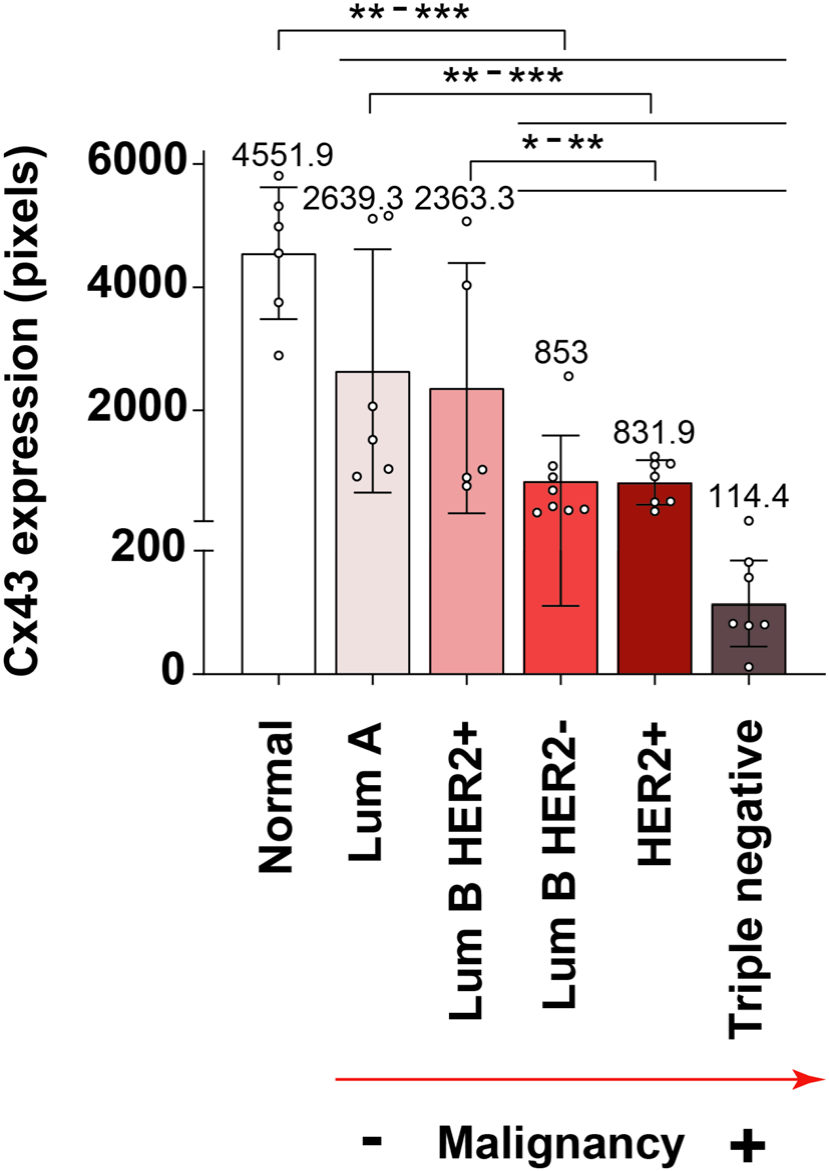
Cx43 is progressively downregulated with increasing breast cancer subtype malignancy. Graph represents Cx43 expression (quantitated as brown area in pixels) relativized to cell density for each patient. Mean+SD; mean values specified in the graph. n=5-8/subtype, each n is represented as a dot in the graph. One-way ANOVA. *$P<0.05$, **$P<0.01$, ***$P<0.001$.

For **SET 1**, Fig. 9 shows bar charts representing Cx43 expression in core needle biopsies of invasive tumours not subjected to chemotherapy or radiotherapy and healthy control tissues, quantitated as brown area in pixels relativized to cell density for each patient. According to these measurements, all breast tumour samples showed significantly lower Cx43 immunostaining than healthy mammary controls. Furthermore, Cx43 signal seemed to be inversely and significantly correlated with breast cancer subtype aggressiveness, being the lowest in the most serious subtype triple negative (ER, PR and HER2 negative), slightly higher in non-luminal HER2+ (ER and PR negative, HER2 positive) and luminal B HER2- (ER and/or PR positive, HER2 negative), and the highest in luminal B HER2+ (ER and/or PR positive, HER2 positive) and in the better-prognosis luminal A (ER and/or PR positive, HER2 negative). In line with these results, Cx43 and/or GJ function downregulation has been extensively described in breast cancer cell lines^27,28^ and primary tumour tissues, suggesting a tumour-suppressing role for Cx43 in primary breast tumours^27,31^. Several studies have also appointed a positive correlation between Cx43 gene and protein levels and ER and PR status, and a negative relationship with HER2 expression^30,45^. In our work, no significant difference was observed regarding Cx43 immunostaining between HER2+ and triple negative breast cancer, as also reported in the literature^29,46^.

**Figure 10:**
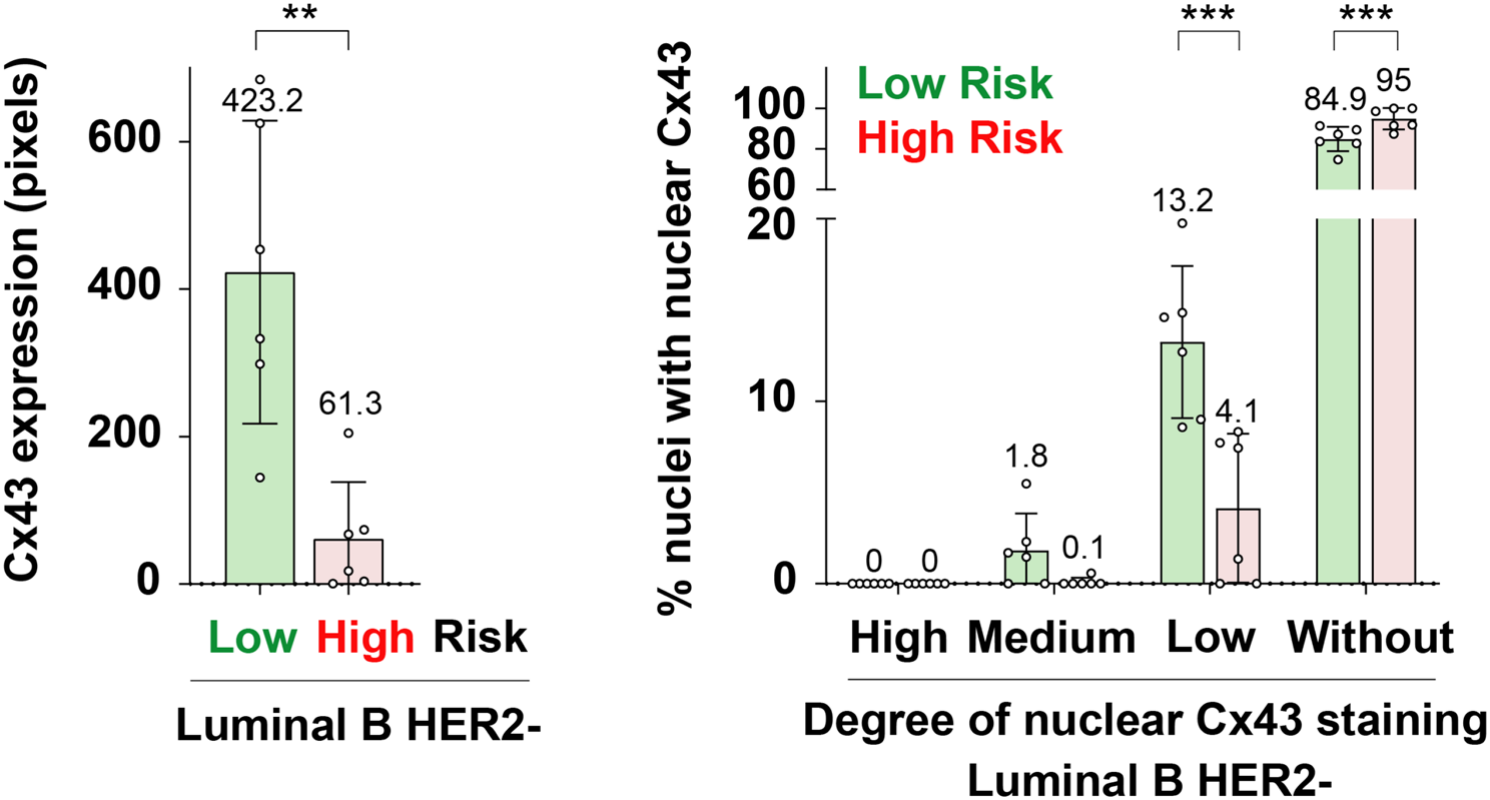
Higher Cx43 is associated to lower cancer recurrence risk in Oncotype DX-tested luminal B HER2- samples. Left graph represents Cx43 expression (quantitated as brown area in pixels) relativized to cell density for each patient. Right graph shows the percentage of cellular nuclei with high, medium, low or no Cx43 nuclear signal. Mean+SD; mean values specified in the graph. n=6/category, each n is represented as a dot in the graph. T-test. **$P<0.01$, ***$P<0.001$.

Next, this analysis was mirrored with images from **SET 2**, comprising invasive breast cancer luminal B HER2- tissues assessed for Oncotype DX Breast Recurrence Score Test. Indeed, Cx43 expression was significantly higher in low cancer recurrence risk tissues than in high risk ones (Fig. 10 left). In order to move one step forward in analysing Cx43 levels in these samples, nuclear Cx43 signal was compared among them, categorizing the cellular nuclei in high, medium, low or without Cx43 staining (Fig. 10 right). The majority of the samples were characterized by null (“without” category) or almost null (“low” category) nuclear Cx43 signal, with high-risk patients having significantly more Cx43-null nuclei than low-risk ones (95% versus 84.9%, respectively). As for nuclei with low Cx43 levels, they were significantly more abundant in low-risk tissues than in high-risk samples (13.2% versus 4.1%, respectively). Regarding nuclei with medium Cx43 staining, low-risk patients tended to present more than high-risk ones, although the difference was not significant (1.8% versus 0.1%, respectively, Fig. 10 right). It became apparent that nuclear Cx43 levels mimicked those of the whole tissue (Fig. 10), with low-risk samples having significantly higher Cx43/more Cx43-positive nuclei (even with low staining levels) than high-risk tissues. In agreement with these data, higher Cx43 transcript and protein levels have previously been significantly associated with better prognosis in breast cancer, namely high overall and relapse-free survival and lower disease recurrence, further reinforcing the potential prognostic value of Cx43 in breast carcinoma^29,30,47,48^. It is key to note that our study is the first to validate BreastAnalyser and to address Cx43 expression in the context of breast cancer samples scored according to the pioneering diagnostic test Oncotype DX, putting forward the possibility of Cx43 as a complementary prognostic marker to take into consideration.

Finally, regarding images from **SET 3**, explicitly included just for the assessment of inter-laboratory software performance, brown signal was subsequently not evaluated at the biomedical level. Nevertheless, it is relevant to note that they present different magnifications and belong to antigens routinely assessed in breast cancer clinical environment. The positive outcome and efficiency of Breast Analyser in this context presents this software as a versatile tool which can also potentially support anatomo- and histo-pathological clinical decisions.

## Conclusions

Breast cancer is the most diagnosed cancer worldwide and represents the fifth cause of cancer mortality globally. Immunohistochemistry can support the oncological diagnosis, therapeutic decisions and biomarker discovery but, currently, its evaluation is often subjective and qualitative due to the lack of suitable image analysis tools. It is also known the heterogeneity of IHC images, intimately related to the high complexity of breast cancer and to the inherent intricacy of pathological images due to differences in tissue processing, staining, image acquisition, etc. The available software tools can be grouped into: (1) generic free tools to analyse the images, in which the user needs some programming skills for an optimal use; and (2) sophisticated algorithms that perform specific task, but are often very time consuming. Some drawbacks of both approaches are the lack of suitable tools to review the analysis before the IHC image quantification, or the need of programming and image analysis knowledge for an optimal use.

Our software BreastAnalyser combines the automatic processing of the IHC image using sophisticated algorithms with a friendly GUI that allows experts to review the analysis before image quantification. The segmentation algorithms implicitly include image variability, saving normalization time. The recognition of DAB-brown signal is almost perfect for all data sets tested (sensitivities higher than 99% and positive predictivities above 95%). The detection of nuclei achieves lower performances strongly depending on the image ($F_1$-score from 0 to 96% for different images). Nuclei classification according to their staining level into categories high, medium, low and without staining is very good (accuracy higher than 93% for SET 2 and SET 1). The elapsed time to automatically process the IHC images was less than one second, and the analysis time of an expert per image depends on the review requirements, but it can be estimated in 3.7 minutes per image, spending only 0.43 ms for brown detection and 0.49 or 0.16 for nuclei detection depending on the method (EBA or RBA) used. The overall perception of BreastAnalyser using the SUS questionnaire reported an average of 85.5 points, which means that the system is perceived as excellent.

Within the biomedical context, BreastAnalyser allowed to perform quantifications of the breast cancer IHC images, leading to the following conclusions: (1) Cx43 is deeply downregulated at the protein level in human invasive breast cancer tissue samples when compared to normal breast, with a tendency to decrease its expression as the subtype malignancy increases. It is minimal in triple negative breast cancer (the most aggressive), followed by HER2+, luminal B HER2-, luminal B HER2+ and finally luminal A (the least aggressive) breast cancer, with the highest levels; and (2) higher Cx43 protein expression is associated to lower cancer recurrence risk in Oncotype DX-tested luminal B HER2- breast cancer tissues. Nuclear Cx43 levels in these samples mimicked those of the whole tissue, with low-risk samples having significantly higher Cx43/more Cx43-positive nuclei (although marginally stained) than high-risk tissues.

BreastAnalyser software can competently support both basic and translational research, offering a straightforward and reliable approach for IHC analysis that overcomes some other available softwares in terms of simplicity and pragmatism. It might also be advantageous for certain tasks of pathological anatomy research, namely automatization of IHC-brown signal quantitation, presenting a potential clinical tool for breast cancer diagnosis and prognosis. Future work includes the validation of BreastAnalyser in more biomedical labs and to test it to quantify positivity on IHC images of other tissues, for which our preliminary visual experiments are very encouraging. From the image segmentation point of view, future work will focus on the improvement of automatic nuclei detection and stratification.

### Ethical approval statement

The present study was conducted following the guidelines of the Declaration of Helsinki and later amendments, and was approved by the regional research ethics committees of the Autonomous Community of Galicia-CAEIG (registry codes 2019/535 and 2015/029, INIBIC lab samples) and the Albacete Integrated Care Management System (registry code 2020/06/071, CRIB lab samples). All tissue donors were adults and informed consent was obtained from them.

## Supplementary Material

The user guide of the BreastAnalyser software is included as a supplementary material.

### Supplementary Information

Supplementary Information.

## Data Availability

BreastAnalyser software and the data sets analysed and annotated during the current study are available in the CiTIUS repository https://citius.usc.es/transferencia/software/breastanalyser.
