# Supplementary material for: Software BreastAnalyser for the semi-automatic analysis of breast cancer immunohistochemical images
Source: Sci Rep. 2024 Feb 6;14:2995. doi: 10.1038/s41598-024-53002-6 (PMC10844656; doi:10.1038/s41598-024-53002-6)
Supplement: Supplementary file 1 — Supplementary Information. [file 41598_2024_53002_MOESM1_ESM.pdf]

Software BreastAnalyser for the automatic analysis of breast cancer immunohistochemical images

Marina Rodríguez-Candela Mateos[1,+], Maria Azmat[2,+], Paz Santiago-Freijanes[1,3], Eva María Galán-Moya[4,5], Manuel Fernández-Delgado[2], Rosa Barbella Aponte[6], Joaquín Mosquera[1,7], Benigno Acea[1,7], Eva Cernadas[2,*], and María Mayán[1,*]

[1]CellCOM research group. Institute of Biomedical Research of A Coruña (INIBIC), Complexo Hospitalario Universitario A Coruña (CHUAC), SERGAS, Spain
[2]CiTIUS Centro Singular de Investigación en Tecnoloxías Intelixentes da USC, Universidade de Santiago de Compostela, Spain
[3]Department of Pathology, Complexo Hospitalario Universitario A Coruña (CHUAC), SERGAS, Spain
[4]Physiology and Cell Dynamics, Centro Regional de Investigaciones Biomédicas (CRIB) and Faculty of Nursing, Universidad de Castilla-La Mancha, Spain
[5]Grupo Mixto de Oncología Traslacional UCLM-GAI Albacete, Universidad de Castilla-La Mancha-Servicio de Salud de Castilla-La Mancha, Spain
[6]Anatomic Pathology Unit, Hospital General Universitario de Albacete,Spain
[7]Breast Unit, Complexo Hospitalario Universitario A Coruña (CHUAC), SERGAS,Spain
*eva.cernadas@usc.es, mmayansantos@gmail.com
+these authors contributed equally to this work

ABSTRACT

Breast cancer is the most diagnosed cancer worldwide and represents the fifth cause of cancer mortality globally. It is a highly heterogeneous disease, that comprises various molecular subtypes, often diagnosed by immunohistochemistry. This technique is widely employed in basic, translational and pathological anatomy research, where it can support the oncological diagnosis, therapeutic decisions and biomarker discovery. Nevertheless, its evaluation is often qualitative, raising the need for accurate quantitation methodologies. We present the software BreastAnalyser, a valuable and reliable tool to automatically measure the area of 3,3'-diaminobenzidine tetrahydrocholoride (DAB)-brown-stained proteins detected by immunohistochemistry. BreastAnalyser also automatically counts cell nuclei and classify them according to their DAB-brown-staining level. This is performed using sophisticated segmentation algorithms that consider intrinsic image variability and save image normalization time. BreastAnalyser has a clean, friendly and intuitive interface that allows to supervise the quantitations performed by the user, to annotate images and to unify the experts' criteria. BreastAnalyser was validated in representative human breast cancer immunohistochemistry images detecting various antigens. According to the automatic processing, the DAB-brown area was almost perfectly recognizable, being the average difference between true and computer DAB-brown percentage lower than 0.7 points for all sets. The detection of nuclei allowed proper cell density relativization of the brown signal for comparison purposes between the different patients. BreastAnalyser obtained a score of 85.5 using the system usability scale questionnaire, which means that the tool is perceived as excellent by the experts. In the biomedical context, the connexin43 (Cx43) protein was found to be significantly downregulated in human core needle invasive breast cancer samples when compared to normal breast, with a trend to decrease as the subtype malignancy increased. Higher Cx43 protein levels were significantly associated to lower cancer recurrence risk in Oncotype DX-tested luminal B HER2- breast cancer tissues. BreastAnalyser and the annotated images are publically available*for research purposes (upon acceptance).

1 Immunohistochemical samples

Immunohistochemistry against Cx43 was performed in 10 µm sections of paraffin-embedded normal and tumoral breast tissues using the OptiView DAB IHC Detection kit (Roche). First, sections were deparaffinated in xylene (10 min X4, PanReac AppliChem) and rehydrated in graded ethanol (ethanol 100%, 96% and 80%, 10 min/each, PanReac AppliChem), finishing in distilled water (10 min). They were then permeabilized for 5 min with phosphate-buffered saline (PBS, MP Biomedicals) supplemented with 0.1% Tween 20 (Sigma-Aldrich) (PBST), and endogenous peroxidase activity was inhibited with OptiView Peroxidase Inhibitor for 10 min (3% hydrogen peroxide, Roche). Sections were washed with PBST (10 min X3 washes) and incubated with Cx43 primary antibody (C6219, Sigma-Aldrich) diluted in PBS for 1 h in a moist chamber. Once the time was over, they were washed again with PBST to remove unbound antibody (10 min X3 washes) and incubated with secondary

antibody (OptiView HQ Universal Linker, Roche) for 10 min. After 2 PBS washes of 10 min each, OptiView horseradish peroxidase (HRP) Multimer (Roche) was added for 8 min. The Linker antibodies bind to the primary ones, and bear multiple non-endogenous HQ haptens, to which the Multimers bind. As a result, the signalling cascade is multiplied on par with background reduction. Sections were washed with PBS (10 min X2) and thoroughly again with distilled water to avoid any unspecific binding. Next, OptiView 3,3'-diaminobenzidine tetrahydrocholoride (DAB, 0.2%, Roche) and OptiView H2O2 (0.04%, Roche) were mixed 1:1 and added for less than 1 min. Samples were extensively rinsed with distilled water to stop the reaction and incubated in water for 30 min (10 min X3). Afterwards, nuclei were stained with Gill III haematoxylin (Merck) for 2 min and samples were washed under running tap water until their colour turned blue. Sections were dehydrated (distilled water, ethanol 80%, ethanol 96% and ethanol 100%, 10 min each), cleared in xylene (10 min), and eventually mounted with Eukitt (Sigma-Aldrich). All reactions were performed at room temperature. Negative controls not incubated with the primary antibody were analysed in parallel.

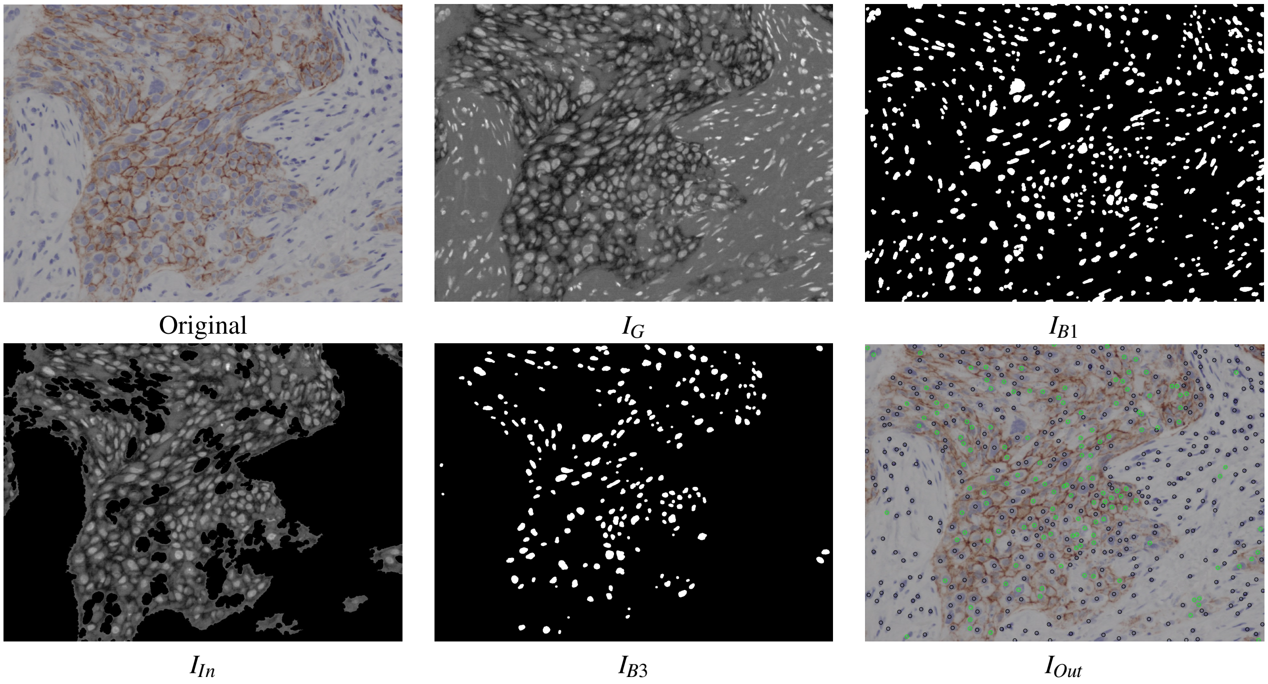

Supplementary figure 1. Examples of the automatic processing of immunohistochemical images using the `RBA` algorithm to detect the nuclei (see pseudo-code in algorithm RBA for the meaning of I_G, I_{B1}, I_{mask} and I_{B3}). The image I_{Out} shows the set of nuclei positions $\mathscr{P}$ overlapped to the original image (in black and green the nuclei without staining and low staining respectively using the classifier).

2 `RBA` algorithm to detect the nuclei

Supplementary figure 1 shows an example of the processing of immunohistochemical images using the `RBA` algorithm to detect nuclei, where I_G is built by adding the I_a and I_b images and normalizing the result to the range [0,255]. The optimal threshold t_G to segment the I_G image is the third threshold ($t_G = t_3$, with $t_G = 132$). To segment the nuclei under the DAB-brown area, the second value t_2 (t_2 is the line 5 of algorithm RBA) is used to inverse threshold I_G developing the image I_{bin} ($t_2 = 88$ for image in supplementary figure 1).The third value t'_3 to threshold the I_{In} image is $t'_3 = 89$.

3 BreastAnalyser user guide

BreastAnalyser is a free software tool for analysis of immunohistochemical microscopic images of breast tissues. It was developed by Centro de Investigación en Tecnoloxías Intelixentes (CiTIUS)[1] of University of Santiago de Compostela (Spain) in collaboration with Instituto de Investigación Biomédica de A Coruña (INIBIC)[2]. BreastAnalyser is a multi-platform software

[1] http://citius.usc.es/
[2] http://www.inibic.es/

written in the programming language C/C++. Due to the variability among sample preparation and acquisition systems in the microscopic images, BreastAnalyser includes a friendly GUI (Graphical User Interface) to visualise, review and modify the recognition of objects of interest before their quantification.

BreastAnalyser works with three types of files: image files, XML files (eXtensible Markup Language) and CSV files (Comma-Separated Values). The image formats supported are the most frequently used like GIF, TIF, PNG, BMP, PPM and JPG. Each processed image generates a XML file which contains the contour and category of each recognized object of interest in an image. This XML file is stored and its graphical content can be superimposed to the image in any moment. The statistical information of the quantitative analysis of every image is exported in a CSV file, such as number of objects of each type, area and diameter of objects, etc. Calculated data can also be saved by BreastAnalyser in order to be reviewed by experts any time later.

The present user guide is organized as follows: Sections 3.1 and 3.2 describe the installation steps for the operating systems Windows and Linux; Section 3.3 describes how to run the Graphical User Interface (GUI) of BreastAnalyser. Section 3.4 describes the configuration of the preferences. Sections 3.5, 3.6 and 3.7 describe the File, Edit and View menus, respectively. The Section 3.8 shows information about the analysis of the results with BreastAnalyser. The section 3.9 describes the application and training of the machine learning model (classifier) to discriminate the stained level of nuclei. Finally, Section 3.10 refers to the Help menu.

3.1 Windows installation

Once the user has downloaded the installation file `setupBreastAnalyser.exe`, a double click on the file will open a dialog window which asks for permission to install a foreign program on your computer, click on “Yes” and a window will show you the Setup Wizard. Clicking on “Next” will start the installation.

The next window allows the user to choose the installation folder, click on “Next” and continue with the installation. A dialog will ask if you want to create a desktop icon. Click on “Next” to continue and confirm if you want to install BreastAnalyser, clicking on “Install”, or cancelling the installation.

Once the installation process has started, a pop up window will show the progress; this overall process may take a few seconds. If you click on “Cancel”, the installation process will be aborted. When the installation has finished the system will inform you and ask for launching the program. Click on “Finish” and check the box *Launch BreastAnalyser* if you want to run BreastAnalyser immediately.

3.2 Linux installation

In Linux, there are several types of packages, and each distribution has its own preferred package format. Ubuntu distributions use the Debian packages (format DEB). The .deb/Debian file containing BreastAnalyser is: `breastanalyser_1.0_all.deb`. It is provided the linux package for Ubuntu 20.04 version.

To install BreastAnalyser, go to terminal, change to the folder where the file `breastanalyser _1.0_all.deb` is located and type the following command: `sudo dpkg -i breastanalyser_1.0 _all.deb` and the system asks you for the administrator password.

If the installation gives errors due to the lack of some packages required by BreastAnalyser, the user must uninstall BreastAnalyser with the command `sudo apt -fix-broken install`, install the required packages with the command `sudo apt install package-name`, and install BreastAnalyser again with the command `sudo dpkg -i breastanalyser_1.0_all.deb`. Then, you can run BreastAnalyser using the following command: `breastanalyser`.

BreastAnalyser can be removed from the computer using the command: `sudo apt remove breastanalyser`.

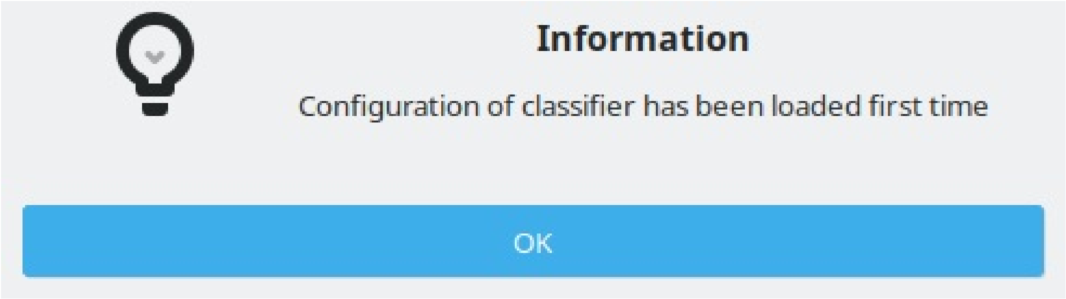

Supplementary figure 2. Pop-up window informing that a default classifier was loaded in BreastAnalyser.

3.3 Run BreastAnalyser

After installing, click on the desktop icon in Windows and a pop-up window informs that a default classifier was loaded (see supplementary figure 2). After clicking **OK** button, the main program window (supplementary figure 3) will be opened. This window encloses a menu bar with several sections, a toolbar containing the main functionality commands of BreastAnalyser, and a window panel where the immunohistochemical images are visualized.

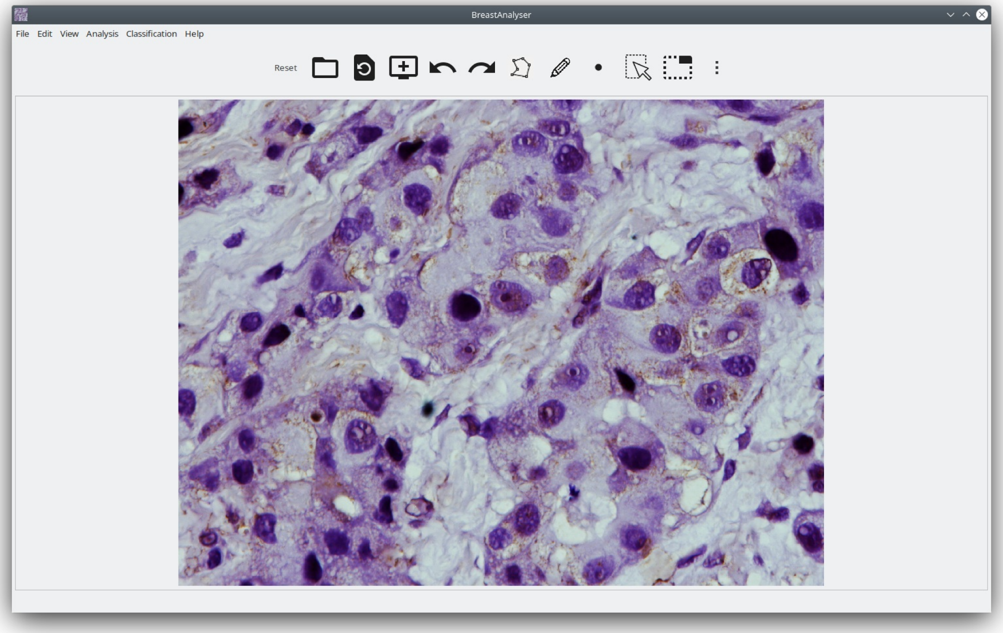

Supplementary figure 3. Main window of BreastAnalyser.

The menu bar lists all **BreastAnalyser** commands and it is organized in six menus:

1. **File**: basic file operations, opening images and XML files, saving the overlays (as xml) and the statistical results (as csv); set and load the working preferences; and exit BreastAnalyser.
2. **Edit**: redo and undo operations, fitting image size and set the image to its original size.
3. **View**: show or hide the processing (lateral) panel.
4. **Analysis**: provides functionalities to calculate the results from several images.
5. **Classification**: provides functionalities to classify cells into their stained level (high, middle, low and without staining) and to train the classifier.
6. **Help**: provides information about the development of the software.

The toolbar provides a faster access to the main functionalities of BreastAnalyser, and contains the following icons (a pop up label shows a short description when the mouse is placed over the icon) from left to right side:

1. **Reset**: clears all the objects drawn on the image.
2. **Open**: opens a dialog to select the microscopic image (see Section 3.5).
3. **Zoom Fit**: fits the zoom of the image window (default behaviour when an image is loaded).
4. **Original Zoom**: resets to the original image size.
5. **Undo**: undoes the last object manually drawn or deleted.
6. **Redo**: performs again the last drawing object.

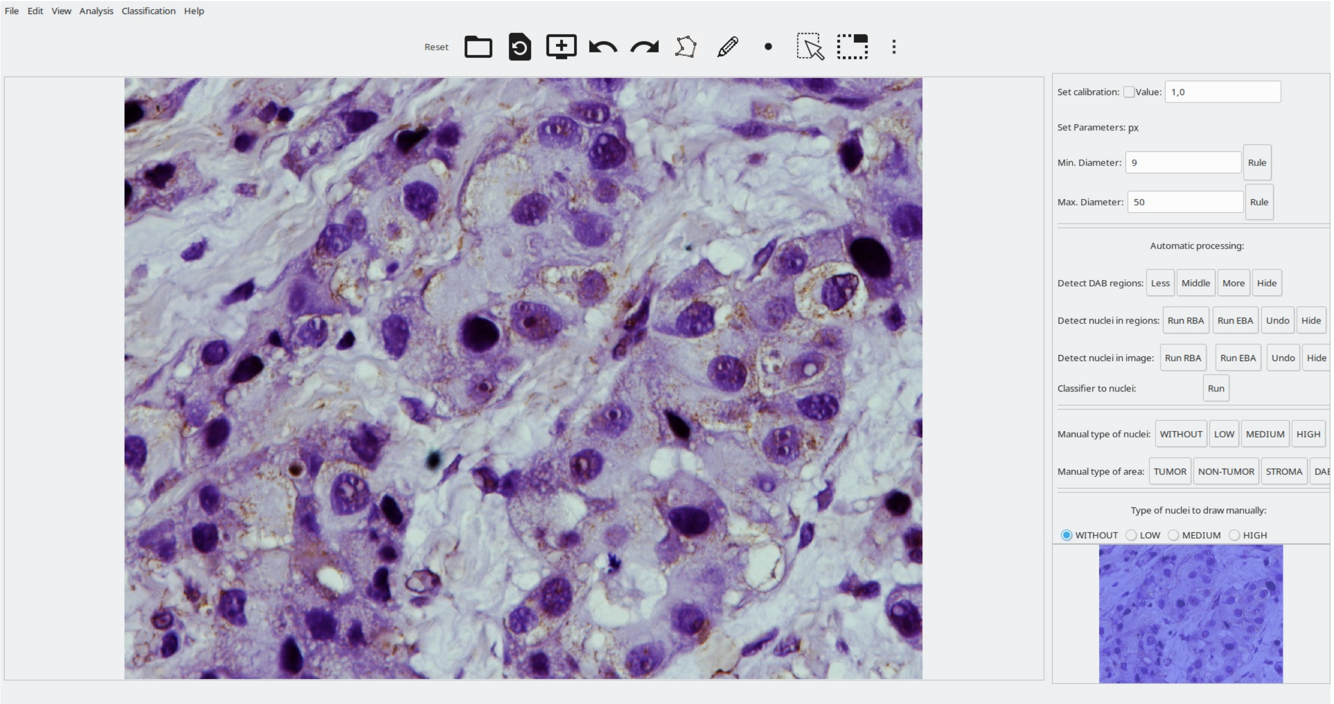

Supplementary figure 4. Main window of BreastAnalyser with the lateral panel.

7. **Draw by points**: marks the object contour by points; use the left button of the mouse to draw them. The contour will be closed by clicking the middle button of the mouse.

8. **Draw freehand**: marks the object contour freehand. To draw a contour press the left button of mouse and keep it pressed while drawing the region. The contour will be closed when the button is released.

9. **Draw points**: when this button is activated, you can mark points (as the software BreastAnalyser considers the cells as points in the immunohistochemical images), on the image clicking the left button of the mouse.

10. **Select**: selects a drawn contour on the image by clicking into the object with the left button of the mouse. To select more than one object, keep the key “Control” pressed while selecting objects. You can also select various objects with the option **Select with rectangle**.

11. **Select with rectangle**: (eleven icon) when this button is activated, you can select many objects drawn on the image drawing a rectangle. For that, click the left button of the mouse and keeping the button pressed, drag the mouse to draw a rectangle and release the left button. The objects inside the rectangle are selected.

12. **Lateral panel**: open the lateral panel that shows the commands to process and analyse the image (see the section 3.7 and supplementary figure 4).

In the following sections the functionalities of the Graphical User Interface (GUI) of BreastAnalyser will be described. In the File menu there are several commands to work with the required files:

1. **Open image**: opens a dialog window to load a new image.

2. **Open image and XML**: opens a dialog window to select the image to load. The contours of the objects in the XML file will be superimposed, when the XML file for that image already exists in the XML directory (the option is set in the preferences) with the same name as the image.

3. **Open XML**: opens a dialog window to select the XML file and loads it on a preloaded image. The user should check that the XML file matches with the image.

4. **Save XML**: opens a dialog window to enter the name of the XML file and chooses the path to save it. By default, BreastAnalyser uses the name of the image with extension xml, and saves the contours in the XML directory set in the preferences.

5. **Export CSV**: opens a dialog window to enter the name of a CSV file in which you want to save the statistical analysis of the image. By default, BreastAnalyser uses the name of the image with extension CSV, and saves the statistical data in the CSV directory set in the preferences.

6. **Preferences**: opens a panel to set the working preferences (see supplementary figure 5), such as working directories, colour and width of lines, calibration of the images, maximum and minimum diameters of the objects of interest. The working preferences can be saved to be available in future sessions, or exported to an XML file.

7. **Reset preferences**: reset all the preferences saved in the system and exit the program.

8. **Load preferences**: allows to load the working preferences from an XML file given by the user.

9. **Exit**: exits the program.

Below, Section 3.4 describes the configuration of the working preferences in BreastAnalyser.

3.4 Set preferences

Selecting "Preferences" in the **File menu**, the window shown in supplementary figure 5 (left panel) will be opened. There, you can configure the following items:

1. **Calibration**: It is the size (in micrometers) of a pixel in the image. The user must set the value for each image to get the measuring of the objects in their real units. Otherwise, values will be given in pixels.

2. **Diameters**: sets the minimum and maximum size of the objects (cells or brown regions) in the images. These parameters can be set in pixels or micrometers depending of the Calibration setting.

3. **Working directories**: sets the default directories for images, XML and CSV files.

4. **Drawing configuration**: sets the colour and width of the lines to draw the contours over the objects and the points.

5. **Colour selection of fundamental categories**: the user can select a colour to represent every category of objects of interest.

6. **Setting or changing configuration**: use buttons **Ok**, **Cancel**, **Save** and **Save As** at the bottom (supplementary figure 5) to set the preferences to this working session, cancel, save the preferences in the system or save the preferences in a xml user file, respectively.

3.4.1 Calibration

The calibration of an image is fixed in the digitalization process, and depends on the magnification used in the microscope and the spatial resolution of the digital camera connected to it. If the user knows the actual calibration of the image, or set of images, he/she should introduce the *Value* in micrometers per pixel. Therefore press "Enter" and check the box after the label *Set Calibration* to confirm that the real calibration value has been added. If it is non-checked, the calibration will be considered in pixels. When this value is fixed, the minimum and maximum diameters will be converted from pixels to micrometers according to the calibration, as you can see in the right panel of supplementary figure 5.

3.4.2 Diameters

The diameter of the objects of interest to recognize varies with the type of object (nuclei or brown regions), the objective of the analysis and the spatial resolution at which the image is acquired. Keeping this in mind, BreastAnalyser provides more versatility allowing the user to choose the minimum diameter of the brown regions (text box after the label *Min. diameter brown regions*), and the minimum and maximum diameters of nuclei (text boxes after the labels *Min. Nucleus Diameter* and *Max. Nucleus Diameter* respectively). The values are set in micrometers or in pixels depending if the calibration is active or not. After setting the values, press the "Intro" or "Enter" key to update. These parameters can also be set on the lateral panel.

3.4.3 Working directories

In this panel the user can introduce the path to set the default directories to store the working files, *Images Folder*, *XML Folder* and *CSV Folder*, as in the right panel of supplementary figure 5. Clicking the button next to the box, users can choose the working directory in an open dialog window (see supplementary figure 6).

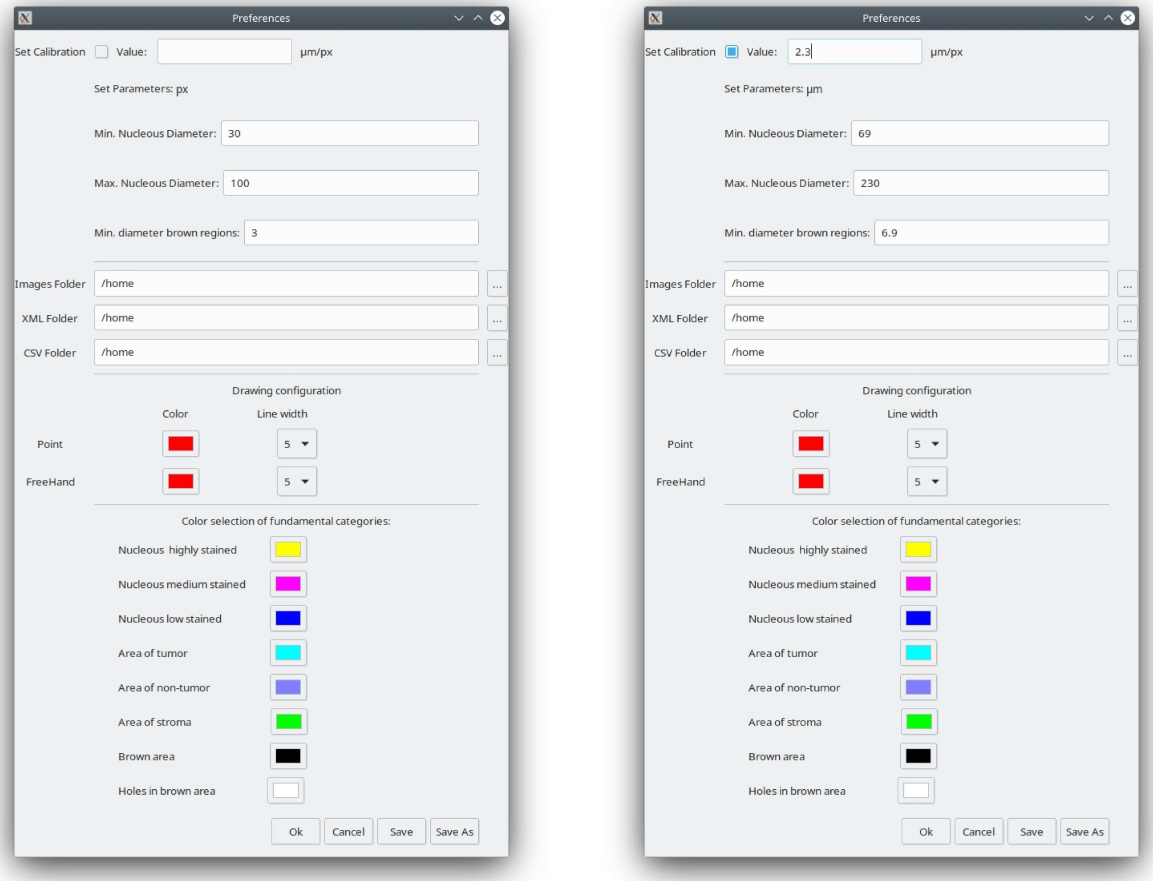

Supplementary figure 5. Setting preferences: (left panel) default configuration; (right panel) after setting the calibration, i.e., the relation between micrometers (μm) and pixels (px)

3.4.4 Drawing configuration

The colour and width of the line and point to draw the object over the image can be changed in this panel. Clicking the coloured button after the label *FreeHand* or *Point*, opens the colour chart dialog shown in supplementary figure 7, choose the colour and press "Select". Clicking on the button "Line width" (right panel of supplementary figure 6) the width of the line can be chosen in a drop-down menu. The colour of every object category can be changed in their own coloured button, below the section *Colour selection of fundamental categories*, with the same process as the *FreeHand* colour.

3.4.5 Setting the configuration

1. Button **Ok**, sets these preferences to the present working session.

2. Button **Cancel**, cancels the operation of setting the preferences. Changes will not be saved.

3. Button **Save**, saves the preferences for present and future working sessions.

4. Button **Save As**, saves the preferences of the present working session and saves them in an XML file selected by the user, which can be loaded using the submenu "Load preferences" of menu "File". This option allows to share the preferences files between users.

3.5 File menu

The commands available in the **File** menu are: **Open Image**, **Open Image and XML**, **Open XML**, **Save XML**, **Export CSV**, **Preferences**, **Load Preferences**, **Reset Preferences** and **Exit**. BreastAnalyser works with three types of files: images, XML (eXtensible Markup Language) and CSV (Comma-Separated Values) files. The supported image formats are the most

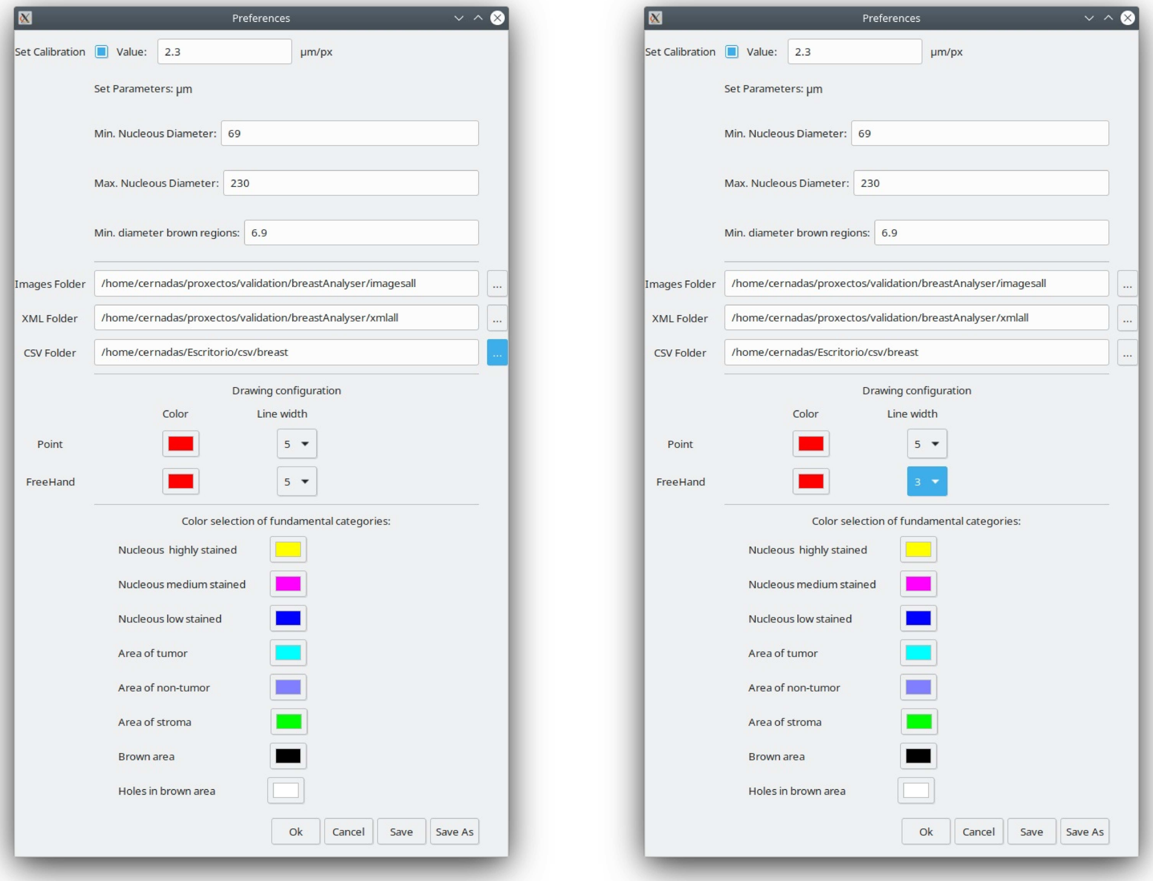

Supplementary figure 6. Preferences panel after setting the working directories (left panel) and after setting the line width of freehand drawing (right panel).

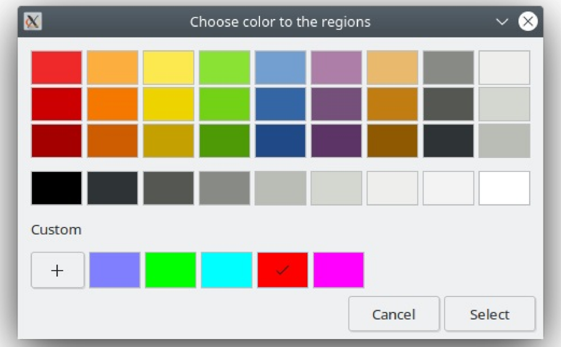

Supplementary figure 7. Chart colour for contour lines.

frequently used ones, such as .gif, .tif, .png, .bmp, .ppm, .jpg, etc. Every image file will have its associated XML file, which will include the contours and categories of the objects of interest, and can be loaded into the software at any time. The CSV file will include the statistical information of the quantitative analysis of every image.

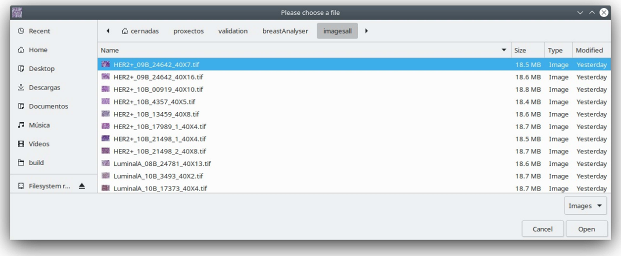

Supplementary figure 8. File chooser dialog to select the image to be loaded in BreastAnalyser.

Open Image opens a dialog (supplementary figure 8) where you can choose an image file from the directory set in Preferences (see Section 3.4) or where you can select another path. Choose the file and click **Open** to load the image in BreastAnalyser. Be aware that the path must not be larger than 256 characters and must not contain rare symbols (“/”, “%”, “ ,”, “*”, “.”, etc).

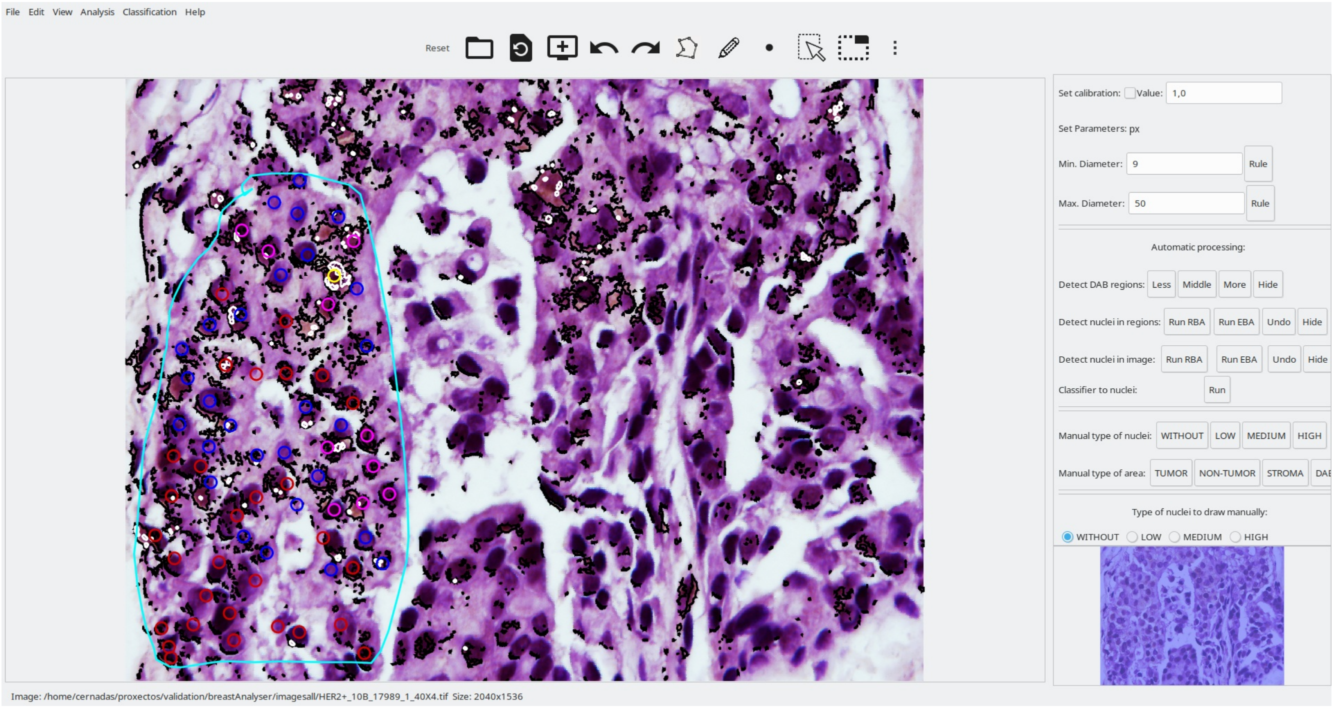

Supplementary figure 9. The image selected in supplementary figure 8 is loaded in BreastAnalyser. The color of the circles represents the stained level of the nuclei: yellow (hightly stained), pink (medium), blue (low) and red (without staining).

Supplementary figure 9 shows the main window of the program with an image loaded and the objects of interest overlapped. When an image is loaded, the lateral panel opens and the state bar at the left-bottom corner of the main window shows the name of image file and its resolution. If the image was already analysed and the XML file was stored, you can load it selecting the submenu **Open XML** and the contours will be shown overlapped to the image, as it can be seen in supplementary figure 9. The user must confirm that the selected XML file is the corresponding one for each image, BreastAnalyser does not check this automatically. Using the submenu **Open Image and XML** (open the window of supplementary figure 8 to select the image), BreastAnalyser will check automatically in the xml directory set in Preferences, if there is a file with the same name as the image. If this is the case, BreastAnalyser will open the image file and overlap the objects of its xml file in a single process (see supplementary figure 9).

Once the contours of the objects have been drawn on the image, the user should open the submenu **Export CSV** to open the window of supplementary figure 10 and set the file name (by default the name of the image with extension csv is used).

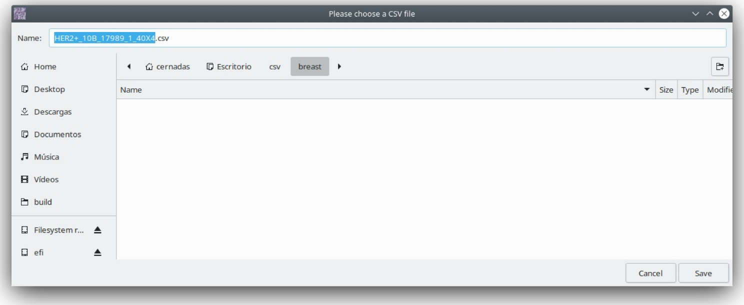

Supplementary figure 10. Dialog to choose the CSV file to store the statistical analysis of the image.

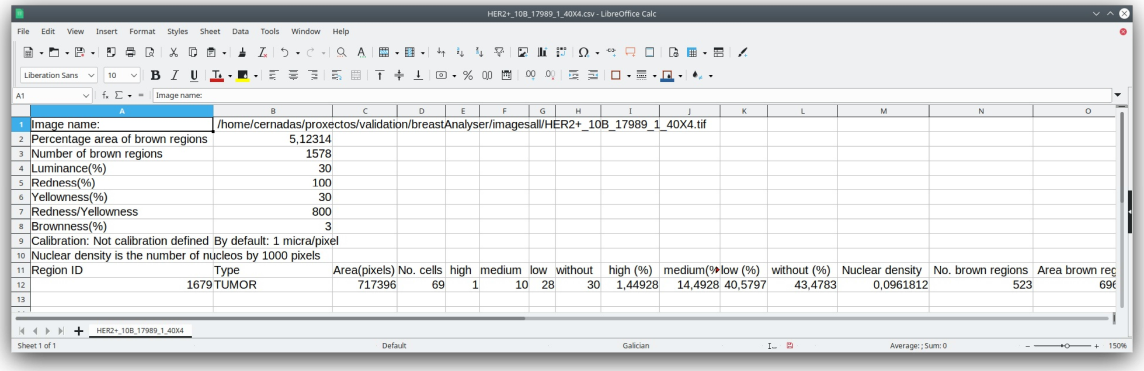

Supplementary figure 11. An example of CSV file of the image of supplementary figure 9 imported in LibreOffice Calc.

The CSV file will store the statistical results of the image in the directory selected in "Preferences". This CSV file contains the following information: 1) the directory path and the calibration; 2) in relation with the brown regions, it contains the number of brown regions and its percentage area, and other statistical measures of the properties of brown regions like the luminance, redness, yellowness, the ratio of redness and yellowness and the brownness; and 3) with relation to the nuclei, the number and percentage of nuclei for each staining level. The information of the CSV file can be loaded in a spreadsheet such as LibreOffice Calc[3], as it can be seen in supplementary figure 11, or in Microsoft Excel.

The **Load preferences** command opens a file chooser dialog to select the XML file preferences to be loaded.

3.6 Edit menu

All the items available within this menu are also available in the toolbar (see Section 3.3). They are: **Undo**, **Redo**, **Fit Image** and **Original Size**.

Fit Image fits the zoom of the image panel and **Original Size** sets the original size of the image. Image zoom can be changed rolling up and down the mouse wheel to increase or decrease the zoom. The portion of the area which is visible in the image window can be seen in the iconized image located at the bottom of the lateral panel (see supplementary figure 12). Keeping the zoom, you can move to another part of the image by two methods: 1) pressing simultaneously the left and right buttons of the mouse over the image panel and displacing the visible area; or 2) pressing the left button of the mouse on the blue square in the iconized image of the lateral panel and displacing the square. Both movements, the visible area in the window image and the iconized image of the lateral panel are synchronized. The overlays of the image are zoomed with the image as well.

3.7 View menu or lateral panel

The only item available is **Processing Panel** (also included in the last icon of the toolbar), which closes or opens the lateral panel of supplementary figure 4. The lateral panel contains the following functionality from top to bottom:

[3] https://www.libreoffice.org/discover/calc/

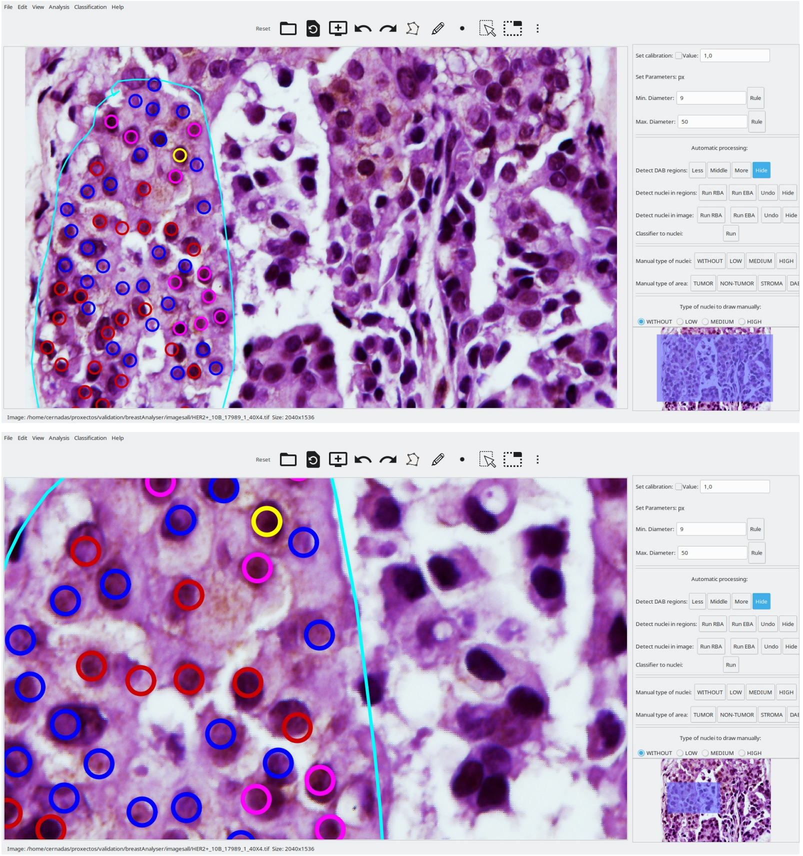

Supplementary figure 12. An example of the zoom functionality in BreastAnalyser.

1. **Calibration** (first line): shows the calibration preferences set in the software or allows to change the calibration options as in the configuration preferences window (see section 3.4).

2. **Diameters** (third and fourth line): shows the minimum and maximum diameters of the nuclei set in the preferences of the program or allows to set these values. The values of the minimum or maximum diameter can be set numerically, using the entry widgets (as it was described in section 3.4), or graphically, using the button **Rule**. When the calibration or diameters are modified, all the overlays of the image are deleted. Using the entry widgets, the value of the diameter would be provided in pixels, if calibration is not activated, or in micrometers otherwise. To choose the diameter graphically, do the following steps: 1) click on the **Rule** button (the button remains activated); 2) draw a line on the image window pressing the left mouse button and when you move the mouse, with the left mouse button pressed, the line is drawing; and 3) when you release the left mouse button, the **Rule** button will become deactivated and the length of the line is put in the entry widget (this value does not appear if the minimum diameter is superior than maximum diameter or the maximum diameter is lower than the minimum one). The changes done in the lateral panel for calibration and diameters are only applied for the current working session. But, if you want to save permanently these changes you must go the preferences submenu (see section 3.4).

3. **Automatic processing**: (from five to nine line) after this label, there is a set of five lines to automatically process the image, which will be described further in section 3.7.1.

4. **Category asignment**: (lines ten and eleven): all these buttons allow to manually set the category of the selected object (see also the 3.7.1 section).

5. **Type of nuclei to draw manually**: the buttons of line 13 allow to select the type of cells that the user wants to draw.

6. **Visualization of results**: when pressing the toggle button **Show Table**, a table containing analysis information is open at the bottom (more details in section 3.7.2). If the table is open, you can click that button (now with the message **Close**

table) to close the table.

7. **Visualization position**: at the bottom of the lateral panel, there is a miniature or icon image of the original image loaded in BreastAnalyser. Over this miniature, a blue square is overlapped, showing the part of the original image which is depicted in the image window. The position and size of this blue square depend on the zoom used in that moment, as can be seen in supplementary figure 12.

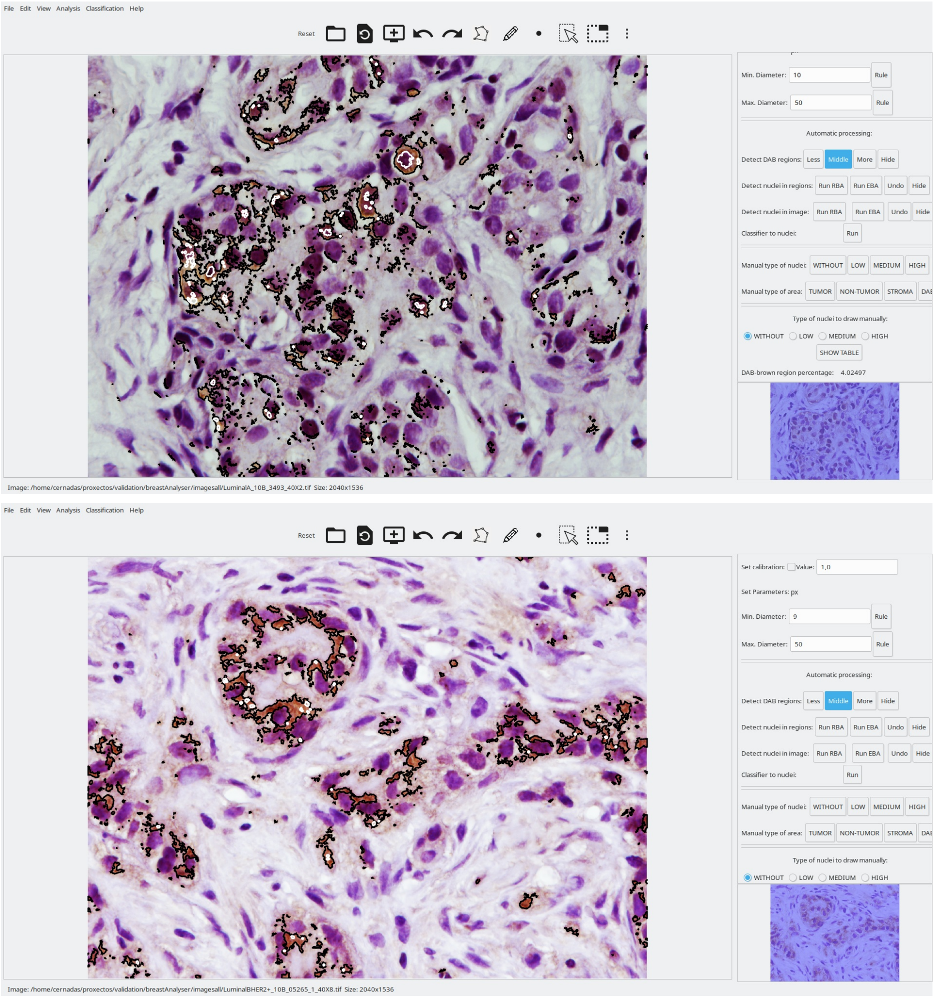

Supplementary figure 13. Examples of the automatic recognition of brown regions using the **Middle** button: the black and white overlays are, respectively, the brown regions and the holes inside the brown regions.

3.7.1 Automatic processing

BrestAnalyser automatically recognizes two types of objects: 1) brown regions (buttons of line 6 after the label *Detect brown regions*); and 2) nuclei of cells (buttons of lines 7 and 8 for detecting the nuclei into a specific area or in the whole image respectively). Finally, BreastAnalyser classifies the nuclei regarding their staining level into four categories: high, middle, low and without staining (button **Run** in line 9 after the label *Classify to nuclei*).

The algorithm included to recognize the brown regions searches for differences in some properties among pixels in the image. It includes three versions of the algorithm, referred as the buttons **LESS**, **MIDDLE** and **MORE** after the label *Detect brown regions*, and the user can test the best result (the algorithm is very fast). Normally, the option **MIDDLE** provides the best recognition. Supplementary figure 13 shows examples of the automatic detection of brown regions. The last toggle button, button **Hide**, allows to hide the contours of the detected brown regions to go on analysing the image, but the regions are not removed. To show the brown regions, click again the **Hide** button.

There are the `RBA` and `EBA` algorithms to detect nuclei in the immunohistochemical images, corresponding to the **Run RBA** and **Run EBA** of the lines 7 and 8 of the lateral panel. Both algorithms can be applied on the whole image or on a region

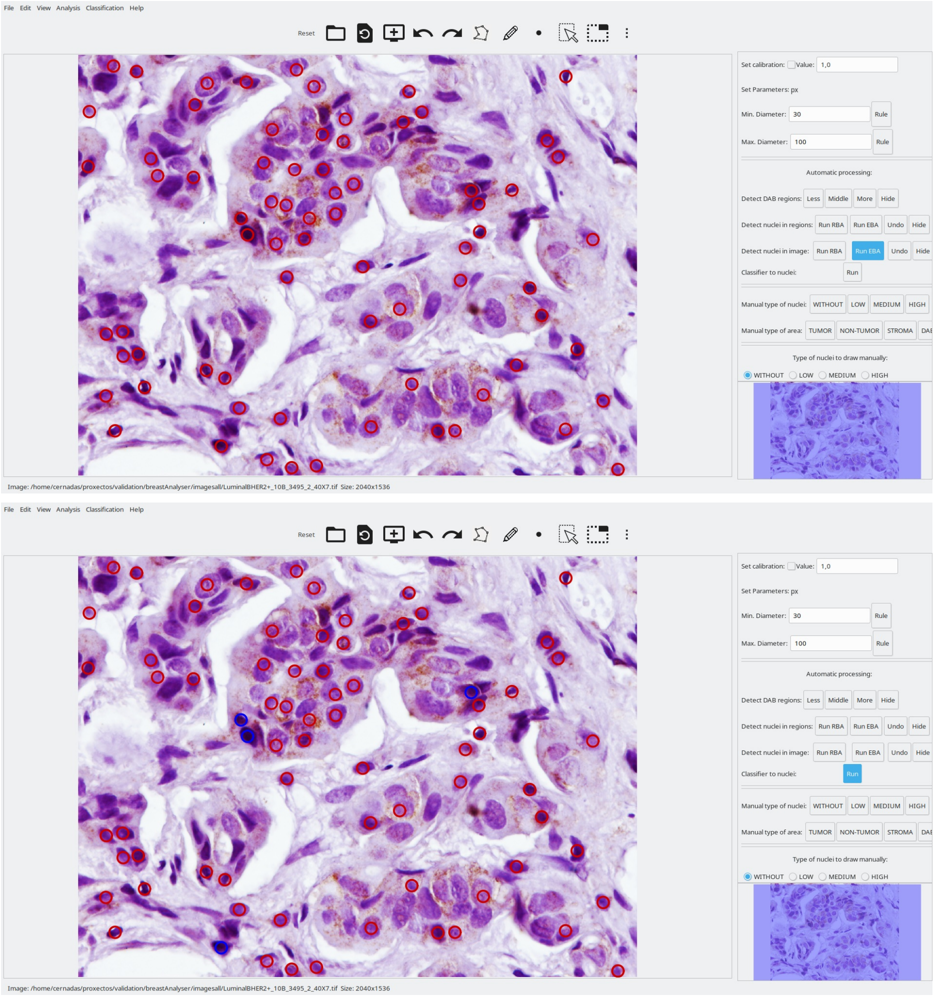

Supplementary figure 14. An example of the automatic detection of nuclei (red circles) in the whole image (upper panel) using `EBA` algorithm and its classification with relation to the staining level (lower panel) – red and blue for nuclei without and low staining respectively–.

defined by the user. The supplementary figures 14 and 15 show examples of the application of `EBA` and `RBA` respectively. To detect the nuclei in the whole image using `EBA`, click the **Run EBA** button after the label *Detect nuclei in image*. The detected nuclei are shown as a point (circle) overlapped on the image (see upper panel of supplementary figure 14). Once the nuclei are detected, you can run the classifier clicking the **Run** button after the label *Classifier to nuclei* in order to automatically set the category to the nuclei. The available categories are **HIGH**, **MEDIUM**, **LOW** and **WITHOUT** for nuclei with high, medium and low level of staining and nuclei without staining respectively (see lower panel of supplementary figure 14). Supplementary figure 15 repeats the process clicking the **Run RBA** for `RBA` algorithm. If the nuclei detection or classification are not satisfactory for the expert, BreastAnalyser provides a friendly GUI to manually review and complete the image analysis. Two ways can be followed:

- **Change the label to one or more nuclei**: change the category of the nuclei detected following the steps: 1) activate the select button in the toolbar and click near the point with the left button of the mouse to select that nucleus (to select more than one nucleus, keep pressed the "Ctrl" or "Control" key); and 2) click one button of line 10 after the label *Manual type of nuclei* to select the suitable category to the nuclei selected.

- **Draw new nuclei with a given category**: to draw new nuclei (not detected automatically) with a given category follow the steps: 1) select the desired category clicking in the radio buttons of line 13 after the label *Type of nuclei to draw manually*, which are **HIGH**, **MEDIUM**, **LOW** and **WITHOUT** any category; 2) active the point draw in the toolbar (ninth button); and 3) click points with the left button of mouse to draw nucleus on the image with the selected category.

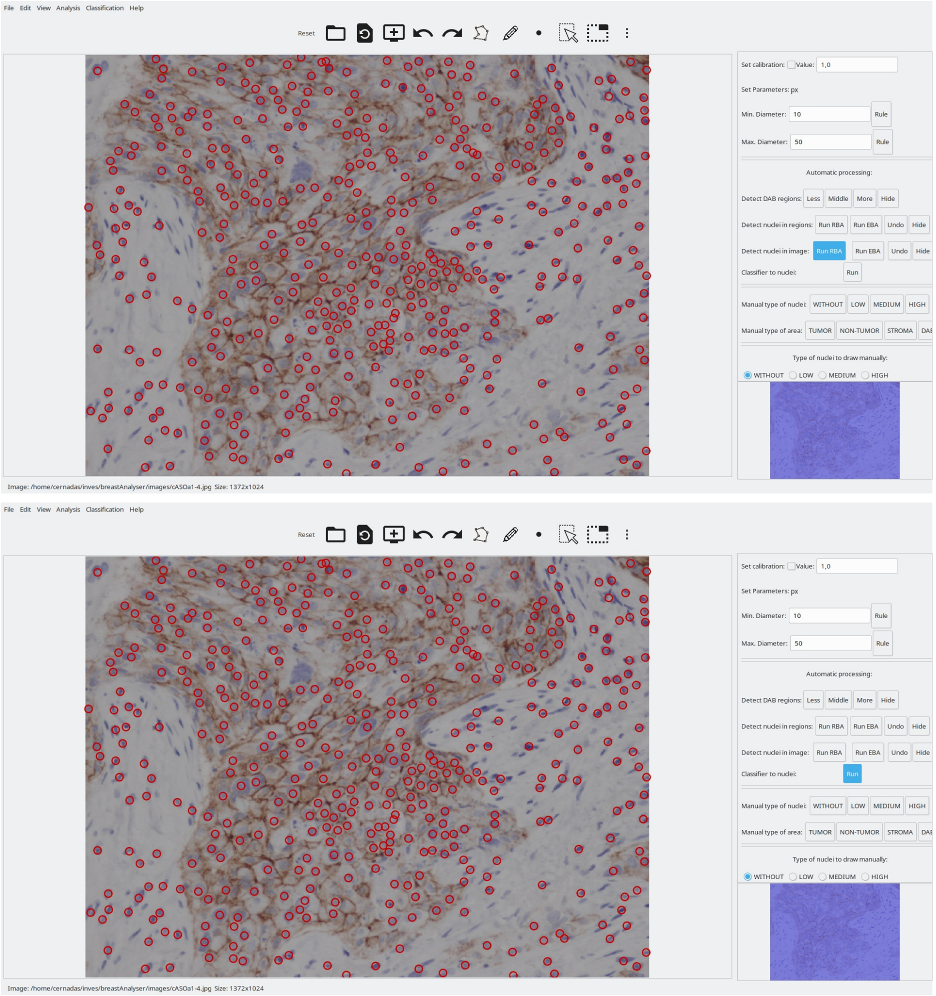

Supplementary figure 15. An example of the automatic detection of nuclei (red circles) in the whole image (upper panel) using `RBA` algorithm and its classification with relation to the staining level (lower panel) – red for nuclei without staining–.

When you want to draw nuclei of other categories, change the category in the radio buttons and repeat the process. It is recommended to choose the radio button **without** when you finish to add nucleus.

Supplementary figure 16 shows another example to detect nuclei inside a user-drawn region. BreastAnalyser also allows to automatically detect the nuclei into a user-drawn irregular region following the next steps:

1. **Draw the regions of analysis**: the user must manually draw the regions of analysis. So, activate the freehand button in the toolbar (seventh or eighth button) and draw one or more regions in the image window using the mouse.

2. **Assign a category to the drawn regions**: activate the button **Select** (tenth button in toolbar) and click inside the region with the left button of the mouse to select the region (see upper panel of supplementary figure 16) and assign the category *tumor*, *non-tumor* or *stroma* clicking in some of the buttons **TUMOR**, **NON-TUMOR** and **STROMA** after the label *Manual type of area* (see line eleven in the lateral panel).

3. **Detect nuclei inside the regions**: detect automatically the nuclei inside the regions representing *tumor*, *non-tumor* or *stroma* clicking the **Run** button after the label *Detect nuclei in regions* in the seventh line of the lateral panel (see the red circles in the upper panel of the supplementary figure 16)

4. **Classify nuclei**: once the nuclei are automatically detected, they must be classified to assign a category to each nucleus (**HIGH**, **MEDIUM**, **LOW** and **WITHOUT** in our case). The lower panel in supplementary figure 16 shows the nuclei of the upper panel after their classification (blue color to nuclei with low staining, pink to midium stained, yellow to nuclei highly stained and red for nuclei without staining).

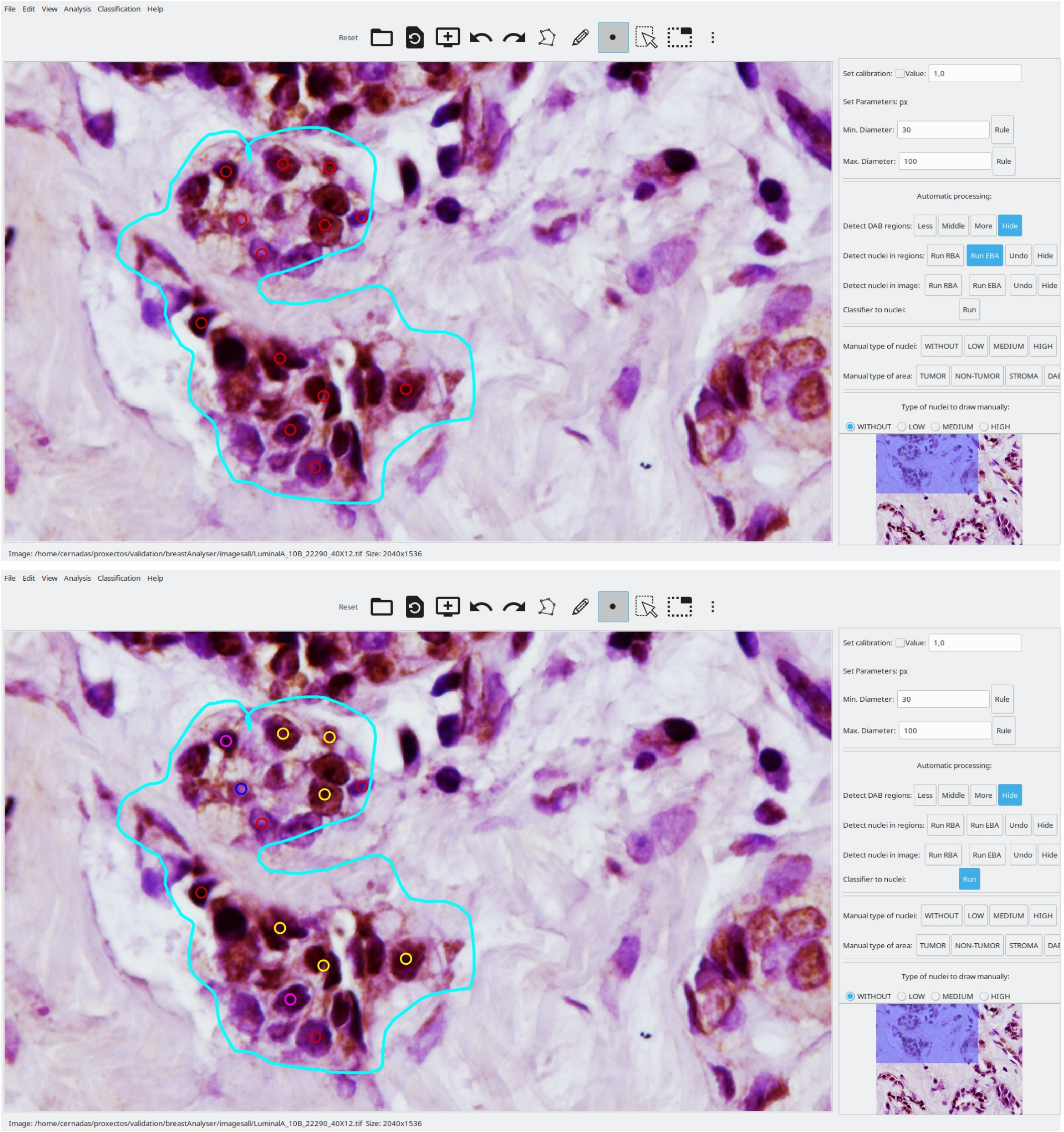

Supplementary figure 16. An example of the automatic detection of nuclei inside the cyan region (red circles in upper panel) and its classification with relation to the staining level (lower panel). The red, blue, pink and yellow colors means without staining and low, medium and highly stained nuclei, respectively.

When the nuclei detection or classification are not satisfactory for the expert, it can be supervised as in the case of the whole image analysis.

3.7.2 Analysis results

After the analysis of an immunohistochemical image of breast following the steps mentioned in the previous section, BreastAnalyser shows and exports the quantitative results for their posterior use (see supplementary figures 17 and 18). The supplementary figure 17 shows the percentage of area occupied by visible brown regions after the label *Brown region percentage* in the lateral panel (if the brown regions are hidden, the percentage will be zero). The supplementary figure 18 shows the percentage of nuclei of each level of staining (without staining, low, midium and high stained). To view this information, press the button **Show Table** of the lateral panel to open a table at the bottom of the main window (see supplementary figure 18) that shows the percentage of nuclei of each type for each region drawn by the user in the image. This table also shows the area, number of nuclei and type of object. Both informations can be exported to a CSV file running the submenu **Export CSV** of menu **File** (see supplementary figure 23).

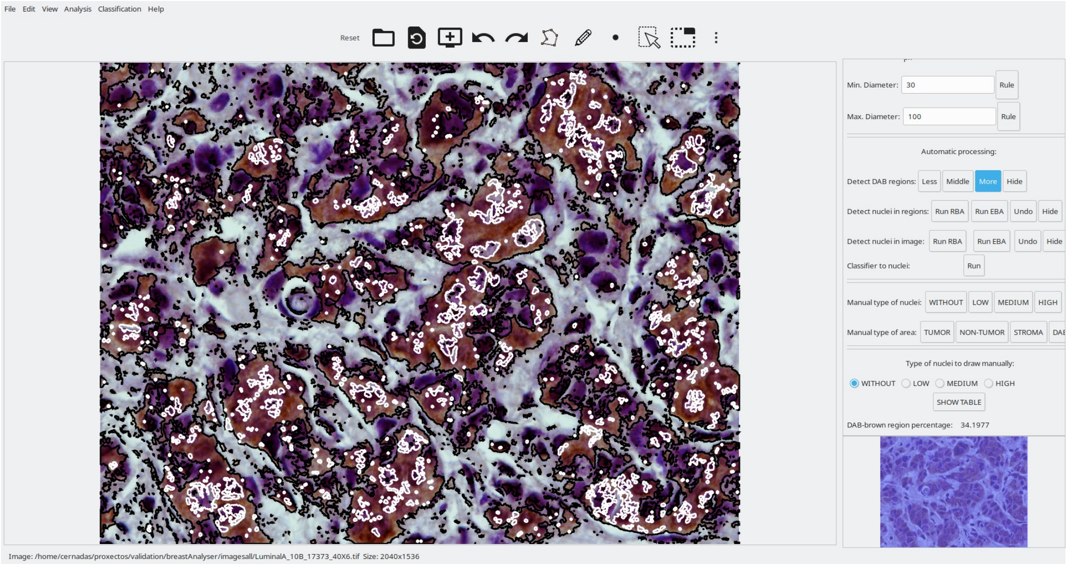

Supplementary figure 17. An example showing the percentage of brown area in the analysed image (see the value after the label *Brown region percentage* in the lateral panel).

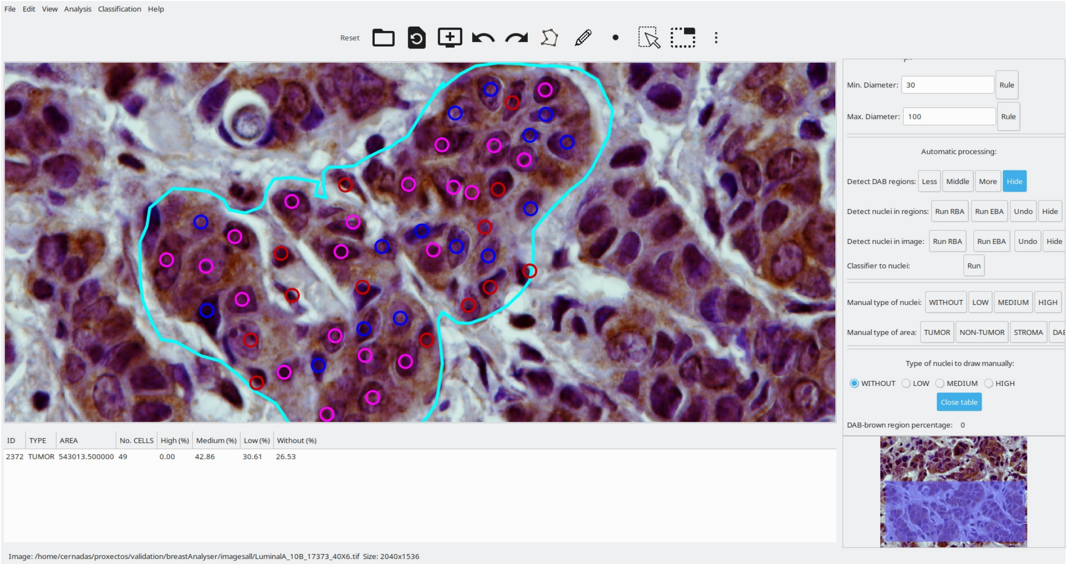

Supplementary figure 18. An example showing the percentage of nuclei of each staining level (low, middle, high and without staining) in each user-defined region.

3.8 Analysis menu

The only item available is **XML File**, which opens the pop up window of supplementary figure 19. Many times the researchers want to accumulate the quantitative results of various images together. For this purpose, you must do the following steps: 1) process each image individually; 2) save the image analysis in an XML file; and 3) run the submenu **XML File** of menu **Analysis** to calculate the jointly quantitative results.

The first line of window of supplementary figure 19, labelled as *XML Folder*, allows to choose the directory of the XML files (by default, it is the XML directory set in the preferences, but it can be changed clicking the three points button). The second line, labelled as *Select XML File*, has the button **Click to select files**. Clicking this button, the file chooser dialogue of supplementary figure 20 is open to choose the XML files used to compute the statistics results. As all file chooser dialogues, a file is selected if you click on the file name with the mouse left button. To choose consecutive files, you click the first file and, keeping the key **Alt** pressed, you click the last one. To choose various files, you click file names keeping the key **Ctrl** pressed. Once the XML files are chosen, click the **Select** button at the bottom of the window. The files selected will appear

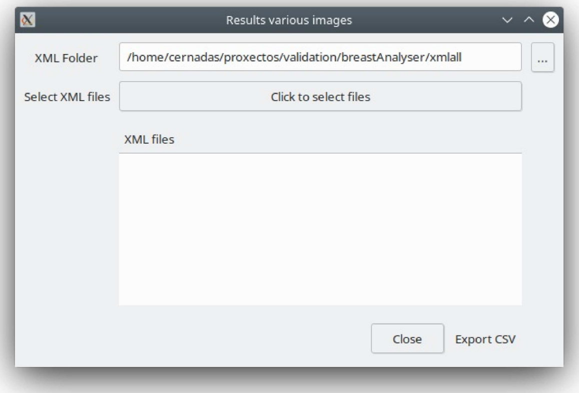

Supplementary figure 19. Window openned when the submenu **XML File** of menu **Analysis** is chosen.

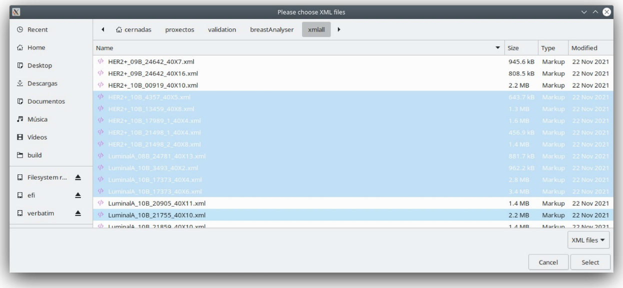

Supplementary figure 20. File chooser dialogue to choose the XML files included in the joint analysis.

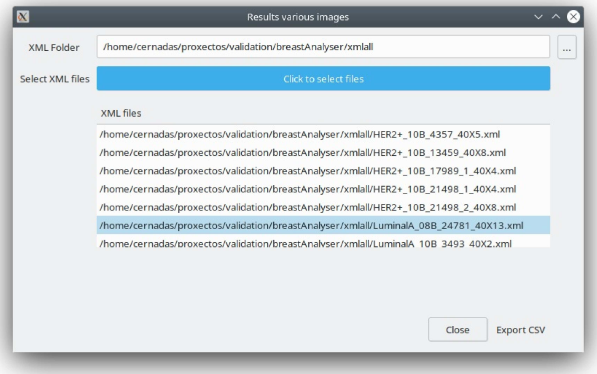

Supplementary figure 21. Window openned when the submenu **XML File** of menu **Analysis** is chosen, after selecting the XML files used in the analysis.

in the supplementary figure 19, as it can be seen in the supplementary figure 21. Then, the user can choose the following operations: 1) cancel this operation pressing the **Close** button at the bottom; or 2) press the **Export CSV** to store the joined statistical results in a CSV file. This last operation opens a file chooser dialogue, which is shown in supplementary figure 22, providing the name `results` for the CSV file. This saving process of the `results.csv` file could require some seconds if there are many XML files to analyse. After saving one CSV file, the window of supplementary figure 19 stays open until the user clicks the **Close** button, in order to do other analysis. One example of the CSV file stored can be observed in the

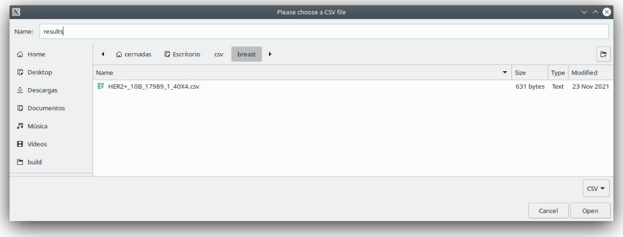

Supplementary figure 22. File chooser dialogue to provide the name of the CSV file.

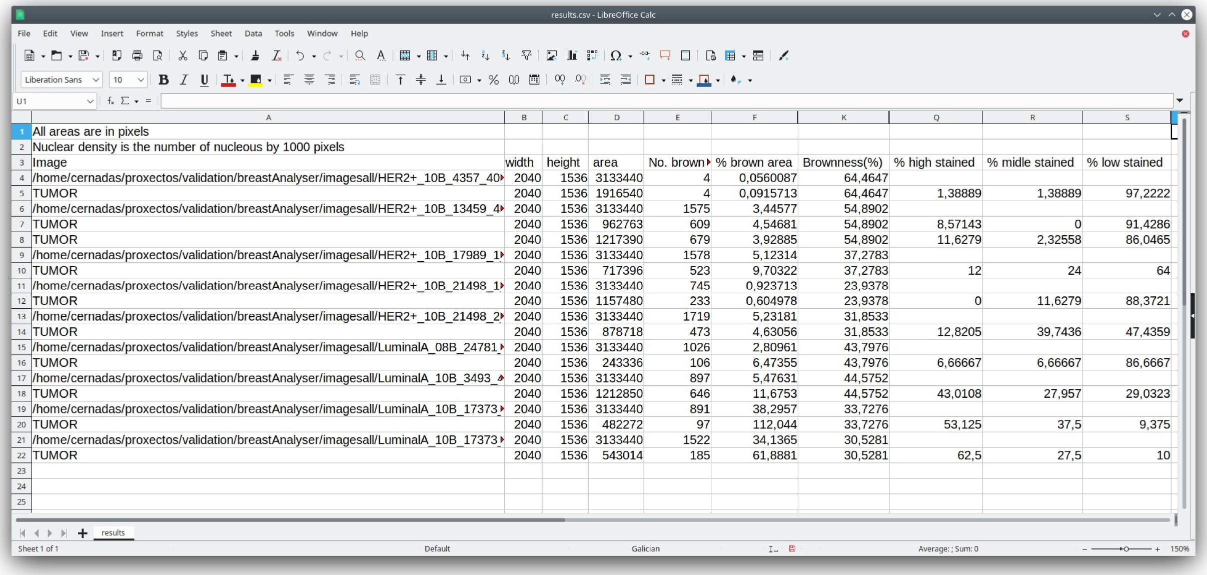

	A	B	C	D	E	F	K	Q	R	S
1	All areas are in pixels									
2	Nuclear density is the number of nucleous by 1000 pixels									
3	Image	width	height	area	No. brown	% brown area	Brownness(%)	% high stained	% midle stained	% low stained
4	/home/cernadas/proxectos/validation/breastAnalyser/imagesall/HER2+_10B_4357_40	2040	1536	3133440	4	0,0560087	64,4647			
5	TUMOR	2040	1536	1916540	4	0,0915713	64,4647	1,38889	1,38889	97,2222
6	/home/cernadas/proxectos/validation/breastAnalyser/imagesall/HER2+_10B_13459_4	2040	1536	3133440	1575	3,44577	54,8902			
7	TUMOR	2040	1536	962763	609	4,54681	54,8902	8,57143	0	91,4286
8	TUMOR	2040	1536	1217390	679	3,92885	54,8902	11,6279	2,32558	86,0465
9	/home/cernadas/proxectos/validation/breastAnalyser/imagesall/HER2+_10B_17989_1	2040	1536	3133440	1578	5,12314	37,2783			
10	TUMOR	2040	1536	717396	523	9,70322	37,2783	12	24	64
11	/home/cernadas/proxectos/validation/breastAnalyser/imagesall/HER2+_10B_21498_1	2040	1536	3133440	745	0,923713	23,9378			
12	TUMOR	2040	1536	1157480	233	0,604978	23,9378	0	11,6279	88,3721
13	/home/cernadas/proxectos/validation/breastAnalyser/imagesall/HER2+_10B_21498_2	2040	1536	3133440	1719	5,23181	31,8533			
14	TUMOR	2040	1536	878718	473	4,63056	31,8533	12,8205	39,7436	47,4359
15	/home/cernadas/proxectos/validation/breastAnalyser/imagesall/LuminalA_08B_24781	2040	1536	3133440	1026	2,80961	43,7976			
16	TUMOR	2040	1536	243336	106	6,47355	43,7976	6,66667	6,66667	86,6667
17	/home/cernadas/proxectos/validation/breastAnalyser/imagesall/LuminalA_10B_3493_4	2040	1536	3133440	897	5,47631	44,5752			
18	TUMOR	2040	1536	1212850	646	11,6753	44,5752	43,0108	27,957	29,0323
19	/home/cernadas/proxectos/validation/breastAnalyser/imagesall/LuminalA_10B_17373	2040	1536	3133440	891	38,2957	33,7276			
20	TUMOR	2040	1536	482272	97	112,044	33,7276	53,125	37,5	9,375
21	/home/cernadas/proxectos/validation/breastAnalyser/imagesall/LuminalA_10B_17373	2040	1536	3133440	1522	34,1365	30,5281			
22	TUMOR	2040	1536	543014	185	61,8881	30,5281	62,5	27,5	10

Supplementary figure 23. An example of CSV file of the analysis of various XML files imported in LibreOffice Calc.

supplementary figure 23. The information saved for each image are: 1) name of image; 2) width and height of the image; 3) area of region or image; 4) number and percentage of brown regions with relation to an image or region; 5) the statistical measures (in %): luminance, redness, yellowness, redness/yellownes and brownness (defined as $(1 - ab/65025) * 100$, the a and b are respectively the intensity of channel a and b in the Lab color space); and 6) the number and the percentage of cells for each staining level in each region.

3.9 Classification menu

As mentioned, BreastAnalyser uses a pre-trained classifier the first time you run it (as it can be seen in supplementary figure 2). Although there is always a classifier trained in BreastAnalyser, it can be trained again with new data in order to improve its performance to predict the staining level of the nuclei. The submenus of the Classification menu from the menu bar are:

1. **Classify**: assign a category (high, medium, low and without) to each nucleus. This functionality can also be executed by pressing the button **Run** after the label *Classifier* in the lateral panel (see the section 3.7.1).

2. **Train classifier**: train the classifier, which is explained below.

The establishment of the staining level of the nuclei (into category high, medium, low and without staining) can be manually set every time using the GUI (as it was described in section 3.7.1). But, BreastAnalyser implements the automatic classification of nuclei. Before applying the classification operation (using the submenu **Classify** or by clicking the button **Run** in the lateral panel after the label "Classifier"), the classifier needs to be trained. The classifier is a supervised machine learning technique, which needs to learn a function to predict the category of the nuclei, which have not been seen before by

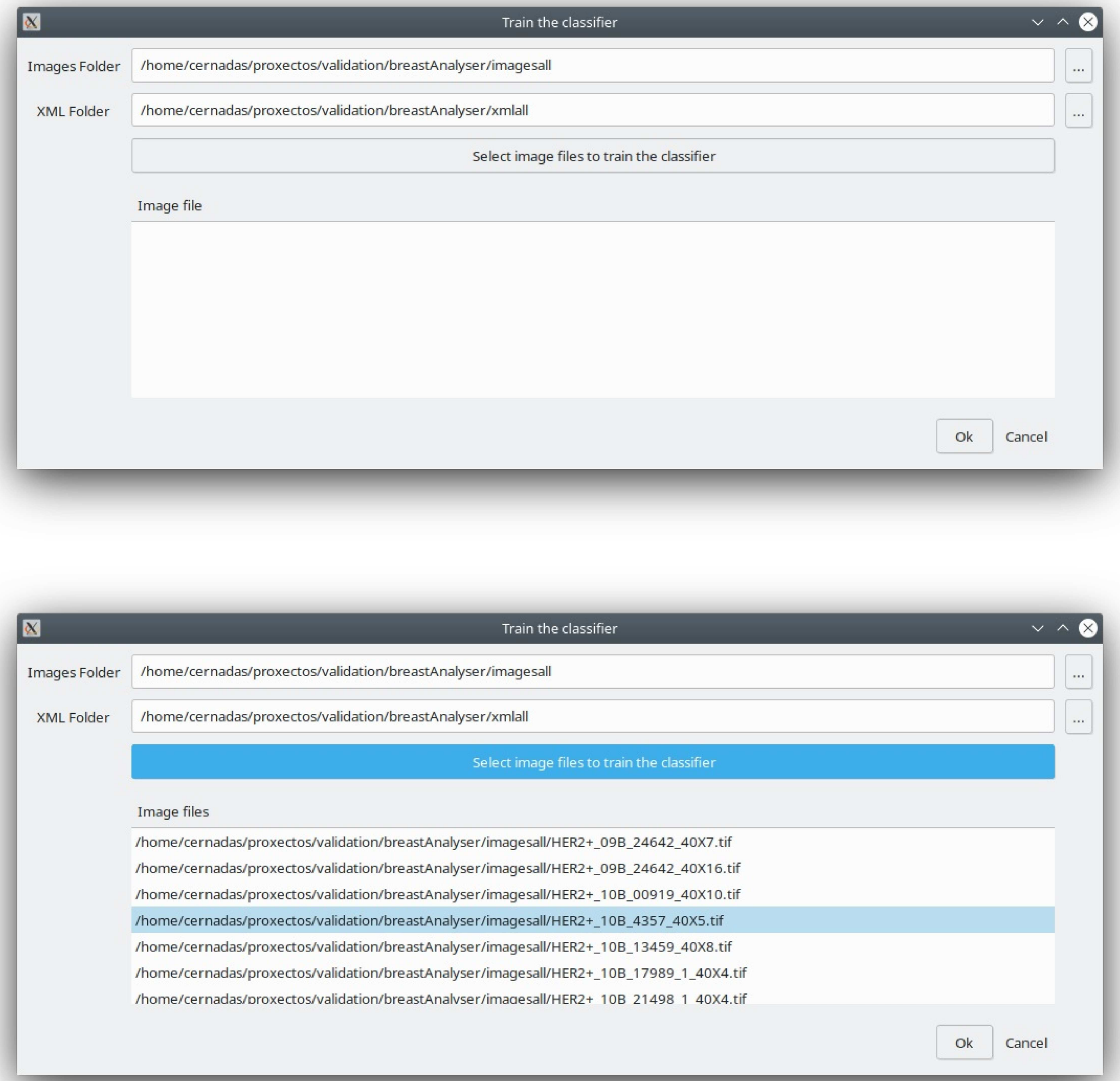

Supplementary figure 24. Window openned to train the classifier: before (upper panel) selection of the image files and after (lower) selecting the image files to train the classifier.

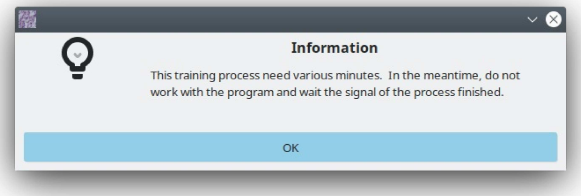

Supplementary figure 25. Pop-up window to inform that the training process needs various minutes to finish.

the classifier. To learn this function it is necessary to provide to the classifier a set of nuclei for which the category is known in a process called training. The image characteristics used to discriminate different categories of nuclei are color features extracted from the image in the position of the nuclei. So, you must manually analyse a limited number of nuclei and save the annotated nuclei overlapped to the image in their corresponding XML files (using the default name, which is the image name with the extension `.xml`) in order to train the classifier. Afterwards, to train the classifier do the following steps:

1. Select the submenu **Train classifier** in menu **Classification**, which opens the window of the upper panel of supplementary figure 24. The first line allows to select the image folder, which is, by default, the image folder specified in the working preferences (see section 3.4). The second line allows to select the XML folder, which is also the folder specified in the preferences by default. As we mentioned in section 3.4 (see the supplementary figure 5), clicking in the button with three points, you can change the image and XML folders.

2. Click the button **Select images files to train the classifier**, which opens a file chooser dialogue as in the supplementary figure 20 to select the image files used to train the classifier. The lower panel of supplementary figure 24 shows the supplementary figure of upper panel after the selection of the image files.

3. Click the button **Cancel** to cancel the training process or the button **Ok** to train the classifier. After clicking the button **Ok**, the pop-up window of supplementary figure 25 informs that the training process needs various minutes depending on the number of images used in the training, the number of nuclei and the complexity of the problem to learn for the classifier). While the BreastAnalyser is trainig its classifier, you can not use it. When the training process is finished, the pop-up window of supplementary figure 26 will be shown.

4. Once the classifier is trained, BreastAnalyser assigns a category to the nuclei clicking the button **Run** after the label *Classifier* in the lateral panel or going to submenu **Classify** in menu **Classification**. Obviously, you can only apply the classification process if there are nuclei detected on the image window.

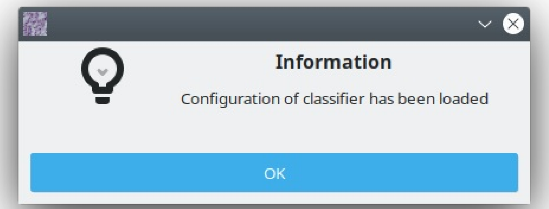

Supplementary figure 26. Pop-up window to inform that the training process has finished and the trained classifier has been loaded into BreastAnalyser.

3.10 Help menu

The **Help** menu is loaded within the menu bar with the submenus **User Manual** and **About Us**. The submenu **About Us** pop-ups a window with a short description of BreastAnalyser and information about its developers and the license (see supplementary figure 27).

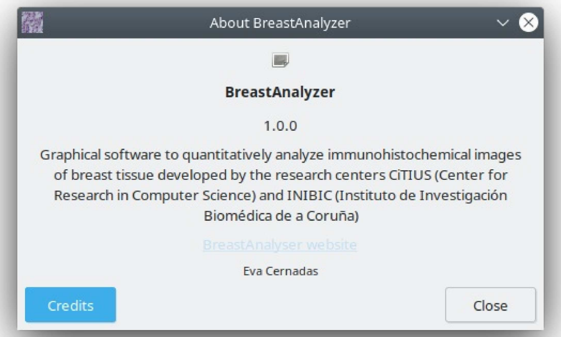

Supplementary figure 27. Pop-up dialog to inform about help.
